# Butyl levulinate production from lignocellulose with mechanistic learning by hierarchical surrogate kinetic modelling

**DOI:** 10.1039/d5gc05536a

**Published:** 2026-01-22

**Authors:** Conall McNamara, Ailís O'Shea, Tiarnán Watson-Murphy, Leandro Ayarde-Henríquez, Thiago De Melo Lima, Stephen Dooley

**Affiliations:** a School of Physics, Trinity College Dublin Dublin 2 D02 PN40 Ireland conall.mcnamara@tcd.ie; b AMBER, Advanced Materials and Bioengineering Research Centre Dublin 2 D02 PN40 Ireland; c Inorganic Chemistry Department, Fluminense Federal University Rio de Janeiro Brazil

## Abstract

This study reports the production of *n*-butyl levulinate (BL) as an advanced biofuel and biomass-derived ester *via* homogenous acid-catalysed butanolysis of four lignocellulosic feedstocks of increasing structural complexity: glucose, cellulose, xylan, and corn cob. A one-pot process valorises the lignocellulosic biomass, improving atom economy, and avoiding multistep derivatisation or protective group strategies. Under optimised conditions, maximum butyl levulinate yields are 49.6 mol% for glucose (170 °C), 43.4 mol% for cellulose (190 °C), 28.8 mol% for corn cob (210 °C), and 8.9 mol% for xylan (210 °C). It is shown that both cellulose- and, for the first time, hemicellulose-derived sugars contributed to butyl levulinate formation, reinforcing the advantage of whole-biomass utilisation over cellulose-focused approaches. A new mass-based yield metric is proposed that accounts for contributions from both carbohydrate fractions, enabling fair performance comparisons across feedstocks. Co-products, including *n*-butyl formate, *n*-butyl acetate, and furfural, are formed in appreciable quantities, offering opportunities for integrated valorisation within a biorefinery framework. Feedstock complexity was found to increase the thermal energy demand required to reach the equivalent conversion. To interpret and generalise the experimental data, a fourth-generation, mass-conserved, semi-mechanistic surrogate kinetic model was developed, based on a learning principle of hierarchical molecular group additivity. The model accurately predicts yields across all feedstocks and is readily adaptable to other lignocellulosic feedstocks in alcoholysis systems. Overall, this work establishes an experimentally validated route to butyl levulinate production that couples high carbon efficiency with a predictive process design tool, advancing the commercial viability of biomass-derived fuels within sustainable, integrated biorefineries.

## Abstract 2

This study reports the production of *n*-butyl levulinate, an advanced biofuel and biomass-derived ester, *via* homogeneous acid-catalysed butanolysis of four lignocellulosic feedstocks: glucose, cellulose, xylan, and corn cob.

## Graphical abstract

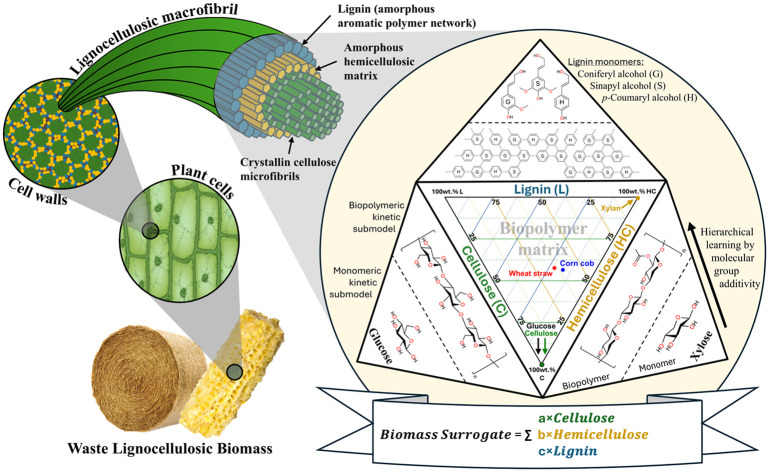

**Green foundation**

1. This work establishes a predictive kinetic framework for one-pot butanolysis of lignocellulosic residues, maximising feedstock utilisation and minimising waste through complete carbon and mass balance analysis.

2. The process uses *n*-butanol as both solvent and reactant, eliminating auxiliary solvents and enabling direct single-step conversion to alkyl levulinates. Model-guided optimisation identifies efficient low-temperature conditions that improve energy efficiency and reduce processing severity. Quantitative coproduct analysis enhances atom economy, with most products directly usable as advanced biofuel blends, reducing waste and improving overall carbon efficiency.

3. Further greening could be achieved by extending the framework to diverse biomass feedstocks, integrating process intensification, and coupling with renewable energy inputs. Future studies may also evaluate heterogeneous catalysts within a techno-economic framework to advance industrial scalability.

## 1. Introduction

The global transportation sector, which encompasses road, air, and maritime transportation, remains heavily dependent on fossil fuels, contributing significantly to anthropogenic CO_2_ emissions, a major driver of climate change.^2^ To mitigate these emissions, biofuels present a promising alternative, as they are carbon-neutral and can be integrated into existing transportation infrastructure without requiring major modifications. However, the commercial-scale production of biofuels faces two primary challenges: i. the competition between food and fuel production and ii. the land-use implications for crop cultivation. A promising solution lies in advanced biofuels, which rely on non-food feedstocks such as agricultural residues and waste materials. Of the advanced biofuel feedstocks listed in the revised Renewable Energy Directive,^3^ lignocellulosic biomass stands out for its high sustainability. This is primarily due to it being a by-product or waste material, which makes its further utilisation both environmentally and economically desirable. The primary obstacle to utilizing lignocellulosic biomass for fuel production is its recalcitrance and the need to remove oxygen from its structure, which is an energy-intensive process that requires complex conversion technologies. Moreover, biofuel production processes must be technically efficient and economically viable, with the product competing in price and quality with incumbent fossil fuels. This techno-economic challenge highlights the need for ongoing research to develop cost-competitive biofuel production processes.^4^

In addition, the study of biofuels addresses sustainability concepts established by Green Chemistry (GC) (*i.e.*, the use of renewable feedstocks and catalysts) and those from United Nations Sustainable Development Goals (UNSDGs) (*i.e.*, affordable and clean energy, sustainable cities and communities, and climate action).^5–7^ Moreover, this approach minimises the CO_2_ environmental footprint by utilizing renewable feedstocks and efficient catalytic systems, while avoiding the use of toxic solvents or hazardous reagents, thereby aligning with GC's principles. In this context, the proposed methodology also integrates the design of processes for energy efficiency and striving for degradation pathways that generate benign by-products, thereby aligning further with GC's sustainability pillars. Overall, our butanolysis strategy supports a circular economy by closing carbon loops and valorising waste into high-value products.

Several thermochemical conversion technologies have been investigated for converting lignocellulosic biomass into biofuels, including hydrolysis,^8^ alcoholysis,^9^ pyrolysis,^10^ gasification,^11^ hydrothermal liquefaction^12^ and hydrothermal carbonization.^13^ Notably, alcoholysis of biomass (*i.e.*, acid hydrolysis in alcohol solvent) has received comparatively less research attention despite its potential to produce alkyl levulinates, which show promise as drop-in biofuels for diesel and gasoline engines.^14^ Additionally, alkyl levulinates have a broad range of industrial uses, including as solvents, platform chemicals, and additives in fragrances and lubricants.^15,16^

In alcoholysis, the carbohydrates in lignocellulosic biomass are converted into esters, specifically alkyl levulinates, while alcohols are transformed into dialkyl ethers.^17^ This process is most promising and sustainable when the alcohol used, such as *n*-butanol, is derived from bio-based sources. *n*-Butanol can be produced through the fermentation of sugars, typically facilitated by bacteria such as Clostridium acetobutylicum.^18^ The resulting reaction mixture predominantly consists of alcohol, dialkyl ether, and alkyl levulinate, along with minor amounts of water and formic acid. The primary products, alkyl levulinate, dialkyl ether, and alcohol, are recognised as viable drop-in components for transportation fuels. However, a significant limitation to their deployment is the blend-wall behaviour exhibited when mixed with conventional fuels, which poses a substantial challenge to the broad commercial utilisation of these biofuels. Howard *et al.*^19^ have suggested that mixtures of alkyl levulinate, dialkyl ether, and alcohol can improve the fuel mixture properties when blended with conventional fuels, opening up new possibilities for optimising fuel formulations. They conducted critical ignition-quality experiments, demonstrating the flexible range of fuel properties that can be tuned by adjusting the relative concentrations of alkyl levulinate, dialkyl ether, and alcohol in the fuel blend. These findings highlight the extensive compositional potential of levulinate ester-based fuels. Additionally, this approach enhances sustainability by reducing derivatives (GC's 8th principle), as the side products are valuable components that contribute to the overall fuel blend.

The butyl-based mixtures of *n*-butyl levulinate (BL), di-*n*-butyl ether (DBE), and *n*-butanol have garnered significant interest as biofuel blendstocks due to their superior ignition and combustion characteristics compared to their ethyl-based counterparts.^20^ To advance the development of butyl-based biofuels, it is essential to quantify the yields of key components such as *n*-butyl levulinate, di-*n*-butyl ether, and *n*-butanol from real biomass feedstocks. Understanding the full range of product concentrations across different feedstocks with distinct chemical complexities is critical, especially given the wide variability in the biochemical composition of lignocellulosic biomass.^21^

From this perspective, the study's kinetic model offers a powerful tool for predicting product yields during butanolysis (and ethanolysis^22,23^), facilitating the identification of the most profitable and carbon-efficient feedstocks and reaction conditions. By modelling how different surrogate biochemical compositions influence yields, the model accounts for the significant biochemical variability of biomasses, even within the same genus, which can be due to factors like climate, geography, and harvest season. Therefore, this approach will help optimise biofuel production by tailoring it to feedstock availability and regional conditions, ultimately improving both the economic feasibility and environmental sustainability of the process.

## 2. Literature review of one-pot alcoholysis processes to produce *n*-butyl levulinate

**Fig. 1:**
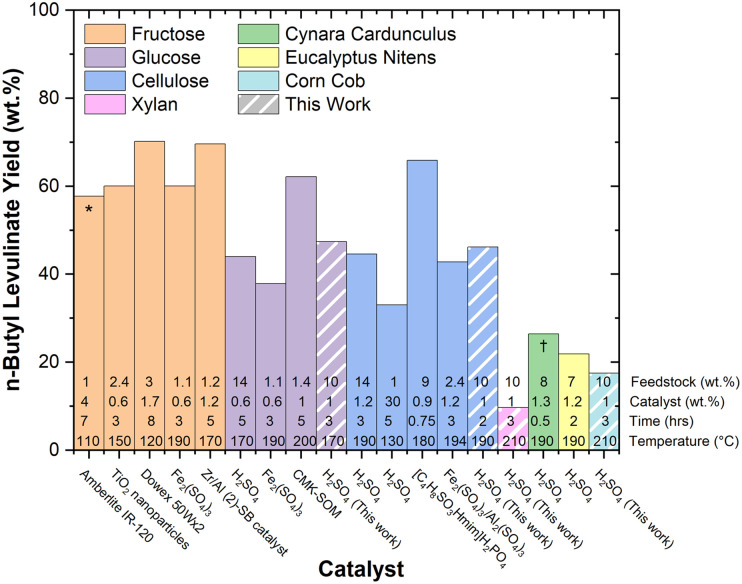
Mass yields of *n*-butyl levulinate obtained from various feedstocks.^25,26,31–40^ Yields are expressed according to eqn (1). Each data point represents a one-pot system containing only butanol, catalyst, and feedstock, with the striped columns indicating this work. The asterisk (*) indicates the use of a co-solvent system comprising γ-valerolactone (GVL) and butanol (30/70 wt%) and (†) indicates that microwave-assisted heating and steam explosion pretreatment were employed.

**Fig. 2:**
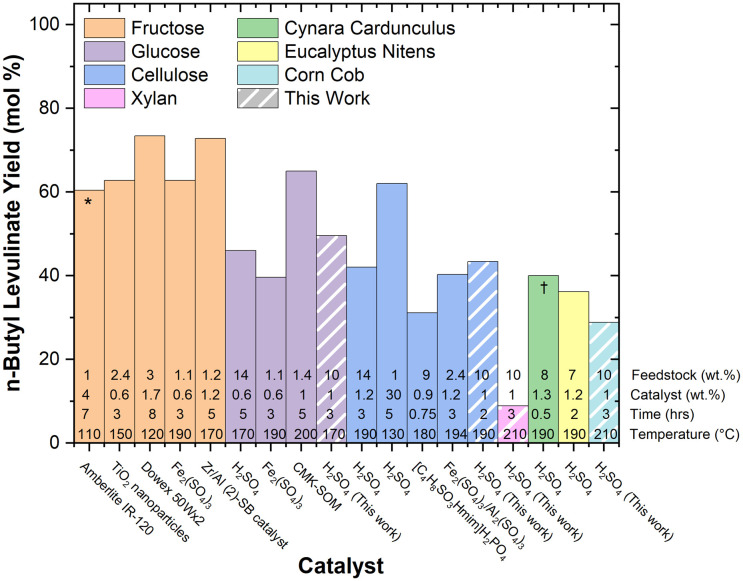
Molar yields of *n*-butyl levulinate obtained from various feedstocks.^25,26,31–40^ Yields are expressed according to the novel molar method described by eqn (2). Each data point represents a one-pot system containing only butanol, catalyst, and feedstock, with the striped columns indicating this work. The asterisk (*) indicates the use of a co-solvent system comprising γ-valerolactone (GVL) and butanol (30/70 wt%) and (†) indicates that microwave-assisted heating and steam explosion pretreatment were employed.

Fig. 1 and 2 present the mass and molar yields of *n*-butyl levulinate from various feedstocks during one-pot butanolysis processes. The yield trends depend on the type of feedstock, in which fructose presented the highest yield, followed by glucose, cellulose, real biomass (cynara cardunculus, eucalyptus nitens, and corn cob), and xylan.

Most studies have focused on investigating the effect of the catalyst on the resulting yield of *n*-butyl levulinate, meaning that both homogeneous and heterogeneous Brønsted and Lewis acids (and combinations thereof) have been investigated intensively, with sulphuric acid (H_2_SO_4_) being the archetypal catalyst. Sulphuric acid has the benefit of being cheap and abundant on an industrial scale. Although this acid may be regarded as corrosive and difficult to recycle, it acts as a strong proton donor and possesses high catalytic efficiency, making it preferred for the conversion of raw biomass to value-added molecules. In addition, the high reactivity allows shorter reaction times and lower temperatures, which can be utilised at low catalyst loading, thereby preventing large waste generation. Compared to heterogeneous catalysts (*e.g.*, zeolites, sulfonated carbon materials, and acid resins), sulphuric acid offers simplicity, enhances atom economy, and can be recovered and reused in closed-loop systems. Sulphuric acid is classified as yellow in the GlaxoSmithKline (GSK) reaction guide and is preferred over other homogeneous acids.^24^ Although using heterogeneous systems allows for a greener process, it is essential to note that these materials often have limitations in recyclability due to the deposition of humin and carbonaceous materials on their surface, thereby decreasing overall activity. Moreover, these materials are required in significantly greater amounts compared to sulphuric acid, thereby increasing the overall process costs. The choice of catalyst and solvent system, along with the simplicity of the one-pot configuration, contributes to the reduction of reaction steps, waste minimisation, and improved process safety, which is in accordance with Green Chemistry's principles. While future efforts may prioritize the transition toward heterogeneous catalysts, the current homogeneous system provides a scalable and atom-efficient baseline for industrial implementation.^5^

We have recently reported that alkyl levulinates (specifically EL) can be produced from xylan and, therefore, hemicellulose fractions of biomass.^23^ This finding challenges the conventional method of presenting molar yields in the alcoholysis literature, where yields for real biomass are typically calculated based only on the cellulose content, as it reveals that cellulose and hemicellulose must be considered to provide a more accurate account of molar yields in biomass conversion processes.^25,26^ However, this methodology overestimates both the molar yield and the overall molecular efficiency of the process, as it neglects the formation of alkyl levulinate from hemicellulose. Similar approaches are commonly found in ethanolysis and hydrolysis research.^27–30^ Such oversimplifications are prone to misrepresent the molecular efficiency of the process and furnish invalid data regarding the atom economy and greenness of the overall reaction. To ensure accuracy and reliability, this practice should be avoided. In the following section, we propose a novel method for calculating molar yields from biomass and present the results in Fig. 2.

## 3. Methodology

### 3.1. Yield reporting methodology

Accurate representation of yield is crucial for analysing biomass conversion processes, particularly in developing emerging technologies. The first part of this study aims to address methodological inconsistencies, overestimations, and, consequently, misunderstandings of yield in the existing literature, and to evaluate options for reporting consistent yields in future studies. The following section presents three methods to compute, and report yield for biomass conversion technologies, each exhibiting advantages and limitations. Depending on the intended application, such as life cycle assessment (LCA), techno-economic analysis (TEA), or mass balance, it may be necessary to report yield in multiple formats (wt%, molar%, and carbon%) to provide a more comprehensive understanding of the process. The equations presented to represent yield use the example of converting lignocellulosic biomass to *n*-butyl levulinate.

#### 3.1.1. Mass yield

The percentage mass conversion of biomass is calculated as the mass yield (wt%) using the eqn (1) ^22^ and is used to report literature yields of *n*-butyl levulinate in one-pot systems in Fig. 1.1
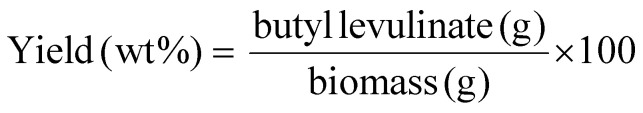


This approach offers several advantages. It is straightforward, requiring no assumptions about the composition of the feedstock, and can be easily applied to various types of biomasses without the need for detailed biochemical analysis or percentage carbon content. However, it has certain limitations because it does not account for the contribution of *n*-butanol in the process. In addition, it does not provide an accurate measure of the molecular efficiency of the reaction. Consequently, it may not fully capture the conversion dynamics or overall effectiveness of the process. Nonetheless, it remains a practical and widely applicable metric for assessing biomass conversion, particularly in preliminary evaluations where simplicity and consistency are prioritised over detailed mechanistic insight.

#### 3.1.2. Molar yield

The percentage molar conversion of cellulose and hemicellulose was calculated using a novel method, as defined in eqn (2) and is used to report literature yields of *n*-butyl levulinate in one-pot systems in Fig. 2:2
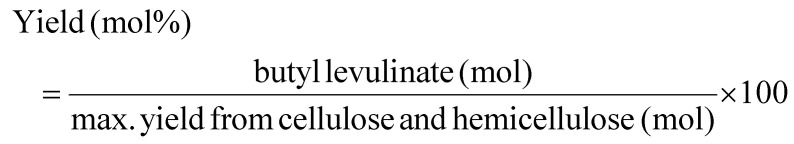


The reaction pathways for these biomass main components are represented by the conversion of the corresponding cellulose (C_6_H_10_O_5_)_*n*_ and dehydrated xylopyranose (C_5_H_8_O_4_)_*n*_ repeating sugar monomers in the presence of *n*-butanol to form *n*-butyl levulinate, water, and formic acid:^23^

*Cellulose*:

3 (C_6_H_10_O_5_)_*n*_ + *n*C_4_H_9_OH → *n*C_9_H_16_O_3_ + *n*H_2_O + *n*HCOOH

*Hemicellulose*:4
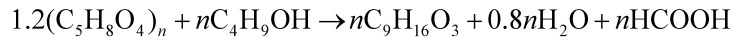


This is a rigorous approach to compute molar yields, as it measures the molecular efficiency consistently with methodologies commonly applied in alcoholysis research.^15,16,41^ However, significant challenges arise when applying this to real-world biomass due to the complex and variable composition of such feedstocks. Unlike pure compounds such as glucose or levulinic acid, for which the number of moles can be precisely defined, biomass contains a mixture of different polymers and associated compounds, making it difficult to represent the associated feedstock in exact molar terms.

The coefficients used in eqn (4) to balance the contribution of an idealised polyxylose chain from hemicellulose underscore the need for further study into the underlying reaction mechanisms. A more detailed understanding would allow a more precise depiction of the full chemical transformations involved. Despite these complexities, this method offers a practical approach for estimating theoretical product yields, with the caveat that assumptions regarding biomass composition must be carefully considered.

#### 3.1.3. Carbon yield

The percentage mole conversion of carbon to *n*-butyl levulinate is expressed by eqn (5):5
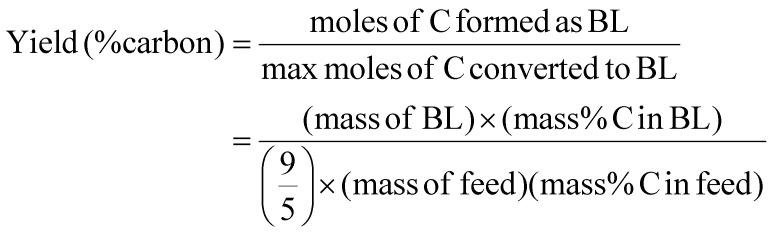


The 9/5 term accounts for the mechanistic fact that five of the nine carbon atoms in *n*-butyl levulinate come from the reacted carbohydrate, while the remaining four come from *n*-butanol. This method is particularly useful when dealing with elemental balances and carbon allocation when performing life cycle assessment calculations, as it allows for computing the amount of carbon converted into *n*-butyl levulinate *versus* the amount diverted into humins and other by-products. Unlike methods relying on biochemical analysis, this approach does not depend on the conversion of xylan to *n*-butyl levulinate, and it accounts for carbon contributions from *n*-butanol.^15^

However, this method has limitations. It is not as straightforward to communicate or interpret compared to simpler yield calculations and requires knowledge of the feedstock's carbon content and the mechanism of product formation. Although this methodology is not commonly used in the alcoholysis literature, it is used for other processes, such as converting biomass-derived feedstocks into hydrocarbons.^42^ This method is particularly valuable for tracking atom flux throughout biomass conversion processes and is therefore valuable for accurate life cycle assessment of such systems.^43^ Furthermore, these calculations are consistent with the principles of green chemistry, particularly those promoting real-time analysis for pollution prevention and the optimisation of resource use through precise carbon accounting.

#### 3.1.4. Space-time yield (STY)

Space-time yield is a process intensification metric that expresses the mass of product generated per unit reactor volume per unit time, as shown in eqn (6). Unlike percentage yields, which reflect molecular or mass efficiency, space-time yield provides insight into overall reactor productivity and is useful for assessing the scalability and economic feasibility of biomass conversion processes.6
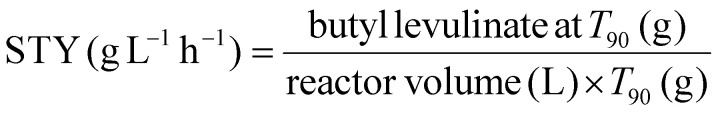


In this study, the space-time yield was calculated using *T*_90_, which is defined as the time required to reach 90% of the predicted maximum steady-state yield of *n*-butyl levulinate. This value was estimated using the hierarchical biochemical surrogate kinetic model,^22,23^ providing a practical approximation of near-steady-state productivity in line with previous studies on biomass valorisation.^44,45^

Space-time yield is especially relevant when comparing reaction conditions, evaluating potential for scale-up, or supporting modelling efforts in techno-economic analysis and life cycle assessment frameworks.^46–48^ However, an essential limitation of the present work is the absence of a comparative reactor optimisation study. The reaction system was developed to access a range of reaction temperatures at a reduced cost, using a PTFE reactor liner with an internal volume of approximately 17.5 millilitres. Only about one-third of this volume was filled during experiments (5 g total of reactants), meaning the reactor configuration is not optimised for maximum productivity. As such, the space-time yield values reported here should not be compared directly to those obtained from fully optimised or continuous reactor systems but instead interpreted within the context of the specific reaction conditions evaluated in this study.

### 3.2. Experimental procedures

#### 3.2.1. Materials

All materials were purchased from commercial suppliers and used without further purification unless stated otherwise. *n*-Butanol (≥99.8%), *n*-butyl levulinate (99%), sulphuric acid (95–97%), anhydrous dibutyl sulfoxide (≥99.9%), and di-*n*-butyl ether (≥99.8%) were purchased from Sigma-Aldrich. Anhydrous d-(+)-glucose (99%), and cellulose (microcrystalline) were purchased from Alfa Aesar. Sodium hydrogen carbonate (≥99.7%) was purchased from Fisher Scientific. Metal autoclaves and 25 mL polytetrafluoroethylene (PTFE) liners are purchased from Xiamen CRTOP Machine Co., Ltd.

Fresh corn cobs (*Zea mays*) were obtained from a local supermarket. After kernel removal, the cobs were cleaned to eliminate any non-lignocellulosic residual. Wheat straw (*Triticum aestivum*) was sourced from a major Irish distributor, Weldon Brothers, located in Deanstown, Swords, County Dublin. The biomasses were dried over several days in an oven at 60 °C until no further mass loss was observed and then milled for 2 hours (h). The resulting powders were sieved using Retsch mesh sieves to collect particle sizes of 100–125 μm.

#### 3.2.2. Biochemical analysis of corn cob

Biochemical analysis of dried ground corn cob was performed by Celignis Analytical according to the National Renewable Energy Laboratory (NREL) procedure for determining structural carbohydrates and lignin in biomass.^49^ The extractives were removed (Dionex ASE-200), and the water-soluble extractives were analysed for sugars. Acid hydrolysis was carried out, and the hydrolysates were analysed on an ion chromatograph (Dionex ICS-3000) to determine the cellulose and hemicellulose content. The hydrolysates were analysed using ultraviolet-visible spectrophotometry (HP 8452A) to determine the acid-soluble lignin content. After that, the hydrolysis residues were then calcined at (575 ± 25) °C to determine the Klason Lignin content. In addition, the ash content was measured after calcination at (575 ± 25) °C for (24 ± 6) h, at a heating rate of 10 °C min^−1^ using a Nabertherm furnace.

#### 3.2.3. Reactive experiments

A feedstock (10 wt%), *n*-butanol (89 wt%), and sulphuric acid (1 wt%) with a total mass of 5 g were added to a 20 mL Polytetrafluoroethylene (PTFE) liner equipped with a magnetic stirrer. The reaction temperature (170–210 °C) and time (0.5–5 h) were the controlled reaction variables. The PTFE liner was placed into a metal autoclave, which was positioned in an aluminium heating block preheated to the desired reaction temperature. After the reaction time, the autoclave was removed from the heating block and allowed to cool to room temperature in air.

Once cooled, the reaction mixture was centrifuged at 5500 rpm for 5 minutes (Benchmark LC-8 centrifuge). Following the separation of the supernatant, the reactions were neutralised by adding an excess of sodium hydrogen carbonate (NaHCO_3_, 50 mg) to prevent potential damage to the gas chromatography column from residual acidity in the solution. An aliquot of the reaction mixture (250 mg) was diluted in dimethyl sulfoxide (DMSO) to achieve a solvent-to-reaction mixture mass ratio of 400 : 1.

Gas chromatography (Agilent 8860, GC-FID) was performed using an Agilent DB-624 column with helium as the carrier gas (constant flow of 20 mL min^−1^). The inlet temperature was maintained at 225 °C, and the detector was set to 300 °C. A 1 μL sample is injected with a split ratio of 16 : 1. The oven program is set as follows: 45 °C for 4 minutes, ramping at 20 °C min^−1^ to 170 °C, and then at 10 °C min^−1^ to 220 °C.

#### 3.2.4. Nuclear magnetic resonance (NMR) analysis

Nuclear magnetic resonance spectroscopy was employed to determine the degree of acetylation of xylan isolated from corn core, a feedstock investigated in this study. ^1^H NMR enables differentiation between two key proton environments associated with the degree of acetylation: hydroxyl (OH) protons, which are replaced by acetyl (COCH_3_) functionalities and readily measurable due to distinct resonances of the protons of the acetyl groups (CH_3_). For each acetylated monomer unit in xylan, an OH moiety is substituted by an acetyl CH_3_ group. Thus, the relative abundance of these proton types provides a basis for quantifying the degree of acetylation.

The degree of acetylation may be expressed in two forms (i) “regiochemical” (*i.e.*, location-based), describing the proportion of hydroxyl groups substituted by acetyl groups, and (ii) “monomer-based”, indicating the average number of acetyl groups per monomer. Both metrics are reported herein.

Approximately 1.0 g of xylan was dried in a vacuum oven at 60 °C for 24 h prior to analysis. NMR tubes (5 mm thin wall, 7″, Fluorochem EU Ltd) and their respective caps were similarly dried. Samples were prepared by dissolving 0.20 g of xylan in 0.80 g of DMSO-d_6_, without the use of a paramagnetic relaxation agent. Spectra were acquired on a Bruker Avance III 400 MHz instrument under the conditions listed in Table 1.

**Table 1:** Experimental parameters used to acquire ^1^H NMR spectra to determine the degree of acetylation of xylan

Parameter	Units	Value
Solvent	—	DMSO-d_6_
Solvent mass	g	0.80
Sample mass	g	0.20
[Cr(acac)_3_]	M	0
Field strength	MHz	400
Flip angle	°	90
Acquisition time	s	4
Delay time	s	60
Time domain points	× 10^3^	64
Sweep width	Hz	8012
Dwell time	Ms	62.4
Scans	—	16
Line broadening	Hz	0.3

##### 3.2.4.1. Location-based degree of acetylation

The location-based degree of acetylation was calculated as the ratio of the acetyl CH_3_ signal intensity to the combined intensity of the acetyl CH_3_ and hydroxyl (OH) signals according to eqn (7).7
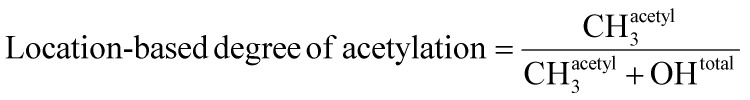
where CH^acetyl^_3_ is the relative concentration of acetyl CH_3_ groups, and OH_total_ is the total relative concentration of hydroxyls.

##### 3.2.4.2. Monomer-based degree of acetylation

To express the degree of acetylation on a monomer basis, two assumptions were made:

1. Each xylose monomer contains either zero or one acetyl substituent.

2. Acetylation accounts for all non-saccharide substituents in the xylan structure.

Assumption (2) is supported by the absence of non-acetyl signals in the NMR spectra. Arabinose, a potential side-chain sugar, would yield overlapping signals with xylose and thus would not affect the validity of the acetylation calculation.

Under assumption (1), each acetyl group corresponds to a monoacetylated monomer bearing one less hydroxyl group. Conversely, unacetylated monomers contain two free OH groups. The relative concentration of unacetylated monomers was therefore calculated as:8
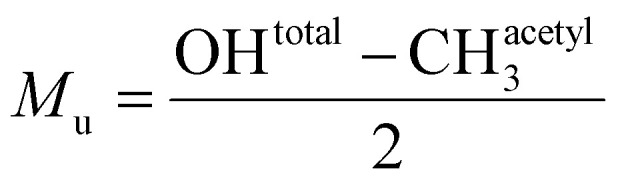
where *M*_u_ is the relative concentration of unacetylated monomers. Accordingly, the monomer-based degree of acetylation was calculated as:9
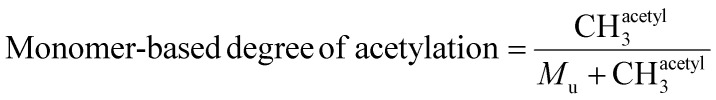


This approach enables accurate quantification of xylan acetylation using simple integrals from ^1^H NMR spectra.

#### 3.2.5. Steady-state conditions

From thermodynamics, a reaction will proceed until the product and reactants are present at concentrations that minimise the Gibbs’ free energy of the system according to:

10 Δ*G*_0_ = −*RT* ln *K*_eq_ = [products]_*t*=*n*_ = [products]_*t*=*n*+1_

where Δ*G*_0_ is the standard state Gibbs’ free energy, *R* is the universal constant of the ideal gas, *T* is the absolute temperature in Kelvin, *K*_eq_ is the reaction quotient at equilibrium (*i.e.*, the equilibrium constant), and [products] is the concentration of species at time (*t*) conditions *n* and *n* + 1.

Whilst measurement of free energy is very difficult, measurement of species fractions is very possible. Specifically, and deliberately learning the time at which steady-state species fractions are reached, with measurement of those species fractions, therefore provides a useful indirect measure of the free energy of the system and an essential physics constraint to the kinetic model. This would (and will) be incorporable into the kinetic molecular if (and when) the requisite molecular thermodynamic quantities of the steady-state species are available.

Establishing the equilibrium composition of a system is essential for validating mechanistic, physics-based kinetic models. To this end, experimental measurement of the mass of all reactants, desired products, and major co-products (*e.g.*, *n*-butanol, *n*-butyl levulinate, di-*n*-butyl ether, *n*-butyl formate, *n*-butyl acetate, furfural, humins, and water) is critical. These data enable estimation of maximum theoretical yields and support the derivation or calibration of thermodynamic parameters embedded within kinetic frameworks.

In batch systems, the concept of steady-state refers to a point at which the concentration (or mass) of key species no longer changes significantly with time, even if true thermodynamic equilibrium is not reached. To define this point objectively, we propose that steady-state is reached when the mass of *n*-butyl levulinate remains constant within ±10% experimental uncertainty over at least three consecutive time points. This definition allows for consistent identification of quasi-equilibrium or plateau phases across experiments, which is particularly important for accurate numerical modelling of complex reaction networks.

#### 3.2.6. Reproducibility and repeatability

Duplicate experiments were performed for every set of reaction conditions, and duplicate gas chromatography analyses were performed for each experiment, thus giving four data points for each reaction condition. The uncertainty bars for the data points on the graphs are the standard deviations of the four data points at those experimental conditions. The uncertainties of the steady-state concentrations and yields are the standard deviations of data points satisfying the steady-state condition set forth in Section 4.5.

### 3.3. Elemental mass conserved hierarchical biochemical surrogate kinetic model

#### 3.3.1. Hierarchical molecular group additivity

**Fig. 3:**
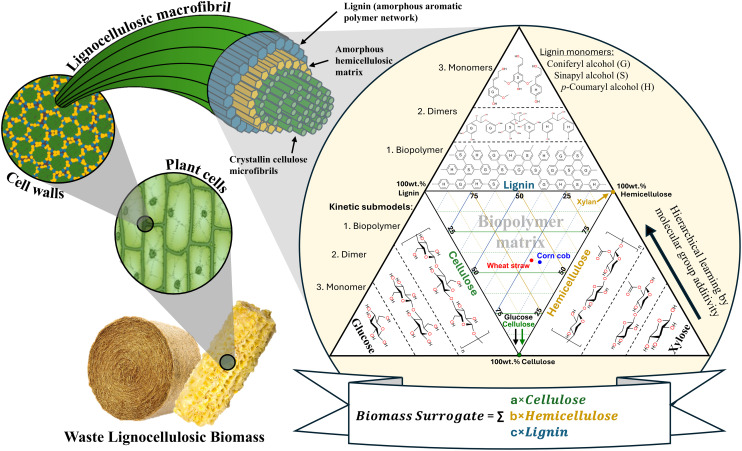
Hierarchical structure and surrogate composition of lignocellulosic biomass, from plant cells to macrofibrils and microfibrils, accompanied by a ternary diagram showing the cellulose, hemicellulose, and lignin contents of various feedstocks, together with representative structural monomers and dimers. This schematic serves as the basis for the biochemical surrogate hierarchical reaction mechanistic kinetic model.

To provide a molecularly self-consistent description of reaction pathways and kinetics, an elemental mass-conserved hierarchical surrogate kinetic model was developed for the sulphuric acid-catalysed alcoholysis of lignocellulosic biomass. The hierarchical mechanism and modelling concept were adapted from previous ethanolysis studies,^50,51^ in which a similar progression from simple to complex substrates was shown to be both chemically valid and computationally robust. The surrogate kinetic model utilises a hierarchical learning framework grounded in the principles of molecular group additivity, as shown in Fig. 3.

The foundational principle of the modelling concept is that the fundamental monosaccharide structures are conserved across all lignocellulosic biomass types. That is, for example, acid-catalysed cleavage of glycosidic bonds proceeds through the same mechanistic steps for cellobiose chains, cellulose chains, and lignocelluloses, just as cleavage of glycosidic bonds proceeds through different but chemically consistent mechanistic steps in xylobiose, in hemicellulose polymers, and in lignocelluloses. This conservation of reaction mechanism allows kinetic parameters to be confidently determined in simpler monomeric systems. These parameters can then be verified and applied to more complex polymeric substrates and similarly extended to compositionally heterogeneous lignocellulosic feedstocks.

Recognition of this hierarchy sets the prospect of a universal mechanistic and reaction kinetic modelling understanding, which encompasses all lignocellulosic biomasses.

The approach is further attractive as it dramatically expands the amount of data available to derive and test kinetic parameters. Computationally, each stage is represented by an independently parameterised sub-model. Taking the cellulose functionalities as an example, kinetic parameters obtained from glucose experiments remain fixed when deriving the cellulose sub-model; similarly, parameters for glucose and cellulose remain fixed when deriving those of the hemicellulose (xylan) sub-model. This structured sub-model by sub-model approach maintains chemical and numerical self-consistency, preventing overfitting and allowing the model to become increasingly predictive as biomass complexity increases.

This hierarchical structure, adapted from the ethanolysis kinetic modelling framework,^51^ ensures that well-defined sub-models form the foundation for more complex systems, providing a chemically justified and computationally stable framework for modelling biomass alcoholysis kinetics.

#### 3.3.2. Biochemical surrogate compositional model

A biochemical surrogate compositional model provides a simplified yet chemically rigorous representation of lignocellulosic biomasses. As conceptualised in Fig. 3, the surrogate concept enables the complex and variable molecular architecture of biomass to be expressed through chemically discrete fractions that are universal across feedstocks, following the hierarchical molecular group additivity principles described above.

The biochemical surrogate framework comprises five principal compositional components, each selected according to the primary biochemical constituents of lignocellulosic biomass and their distinct reaction chemistries. These components are outlined below.

##### 3.3.2.1. Cellulose fraction

Cellulose represents a major fraction of lignocellulosic biomass. It is composed of glucose monomers joined by β-glycosidic linkages of varying chain length, typically of the order of several hundred units.^52^ As cellulose is readily obtainable and structurally well-defined, it is employed directly as the biochemical surrogate component, with glucose and cellobiose defined as the fundamental structural units for mechanistic study within the molecular group additivity framework.

##### 3.3.2.2. Hemicellulose fraction

Hemicellulose, which accounts for roughly 20–35 wt% of dry biomass depending on feedstock composition, is a heterogeneous mixture of polysaccharides composed mainly of pentoses, hexoses, uronic acids, and acetyl groups. Among these, xylans, β-1,4-linked xylopyranose polymers functionalised by acetyl, arabinosyl, and 4-*O*-methylglucuronosyl groups, are predominant in hardwoods and herbaceous feedstocks.^53^ Due to the structural variability of hemicellulose across biomass types, xylan is commonly adopted as a representative model polymer. At the molecular level, xylopyranose and xylobiose capture the essential monosaccharide and β-glycosidic bond motifs used to describe the mechanisms of deacetylation, pentose release, and furfural formation.

##### 3.3.2.3. Lignin fraction

Lignin is structurally more diverse than the carbohydrate polymers, comprising various substituted phenylpropanoid units linked through ether and carbon–carbon bonds. While direct kinetic modelling of lignin functionalities lies beyond the scope of the current work, as we do not see specific lignin-derived products other than humin species, its primary structural motifs, syringyl, guaiacyl, and *p*-hydroxyphenyl aryl ethers, are identified as the fundamental functionalities for detailed mechanistic study in future model expansions.^54^

##### 3.3.2.4. Extractives fraction

Extractives are the non-structural, solvent-soluble constituents of biomass, comprising waxes, fats, resins, gums, phenolics, and other low-molecular-weight organic compounds such as terpenes and free sugars.^55^ These compounds are typically soluble in non-polar or moderately polar solvents and vary substantially between biomass types. Although generally present in minor quantities, extractives can influence alcoholysis kinetics by adsorbing to catalyst surfaces or stabilising reaction intermediates. Removal of aliphatic extractives has been shown to enhance yields of acidolysed xylo-oligosaccharides, suggesting that these compounds may inhibit acid-catalysed reactions by shielding or neutralising active sites.^56^ In this study, extractives are not represented explicitly and are incorporated into the aggregated “Remaining Mass” fraction.

##### 3.3.2.5. Ash fraction

Ash represents the inorganic mineral content of biomass and is primarily composed of oxides, silicates, carbonates, and phosphates of alkali and alkaline-earth metals such as potassium, calcium, magnesium, and sodium, along with smaller quantities of silica and trace elements.^57^ While not directly reactive in organic alcoholysis, these minerals can modify catalytic performance by neutralising acid sites or forming bisulphate salts that reduce the concentration of free protons.^58,59^ Given this study's focus on organic reaction pathways, the ash fraction is not modelled independently but is aggregated within the “Remaining Mass” submodel.

##### 3.3.2.6. Remaining mass fraction (current study representation)

In the present study, direct evidence of low-molecular-weight products arising from lignin or extractive degradation under sulphuric acid-catalysed butanolysis was not observed. Therefore, lignin, extractives, and ash are collectively represented within an aggregated “Remaining Mass” fraction. This sub-model provides a simplified means of accounting for non-carbohydrate components, encompassing non-catalytic pathways that may lead to humins or soluble unidentified products, as observed experimentally in both butanolysis and ethanolysis studies.

By explicitly defining the surrogate composition and independently parameterising each fraction's mechanism and kinetics, the model reduces parameter uncertainty, preserves chemical self-consistency across the hierarchy, and enhances predictive reliability when extrapolating from well-defined model substrates to compositionally heterogeneous and variable lignocellulosic feedstocks.

It is emphasised that the present work focuses on the development and validation of submodels for glucose, cellulose, and hemicellulose, with all remaining biomass constituents treated in aggregate form for simplicity and computational tractability, given the experimental evidence reported herein. Subsequent studies should incorporate explicit lignin, extractive, and ash submodels to assess their influence on alcoholysis kinetics, humin formation, and product distributions. Future extensions should also include xylose as a pentose feedstock analogue to glucose, together with its corresponding dimer xylobiose, to provide a fully generalised representation of both cellulose- and hemicellulose-derived reaction pathways within the hierarchical surrogate kinetic framework.

#### 3.3.3. Chemical reaction mechanism

##### 3.3.3.1. Reaction conditions of the butanolysis system

**Fig. 4:**
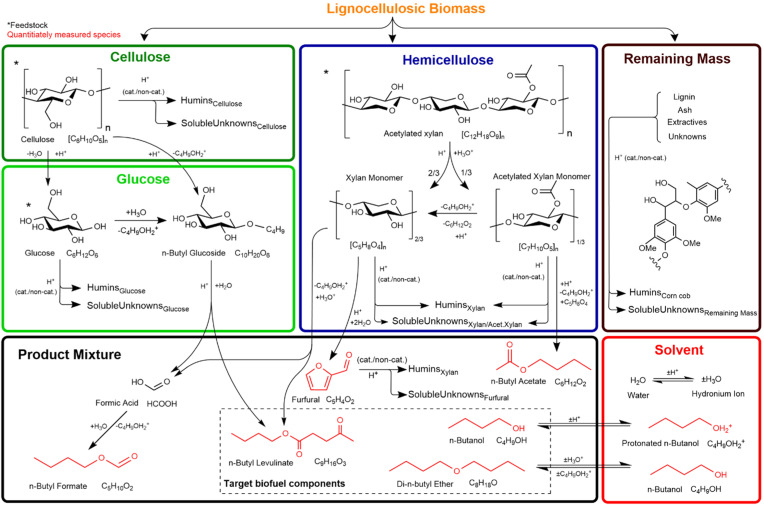
Hierarchical biochemical surrogate kinetic model, illustrating the proposed reaction mechanism and hierarchical sub-models. Associated reaction equations are provided in Table 2. Humins are insoluble by-products formed during alcoholysis, while unknowns, determined by mass balance, represent other soluble species of unidentified structural composition. The representative lignin structure is adapted from Wang *et al.*^1^

The mechanisms of product formation are summarised in Fig. 4, and the associated reaction set and rate constants are listed in Table 2. The hierarchical biochemical surrogate kinetic model reproduced the concentration–time profiles of the eight most abundant liquid products with high accuracy, confirming its capability to capture the dominant pathways and kinetics of the butanolysis network. Under the conditions studied, the butanolysis system exhibited reaction pathways, intermediate formation, and kinetic trends analogous to those previously reported for ethanolysis,^22,23^ consistent with the shared acid-catalysed cleavage and rearrangement steps.

**Table 2:** Reactions incorporated into the hierarchical biochemical surrogate kinetic model, with corresponding empirically derived kinetic parameters (f = forward reaction, r = reverse reaction). Colour coding denotes sub-models: red = solvent, light green = glucose, dark green = cellulose, blue = xylan, and black = remaining mass

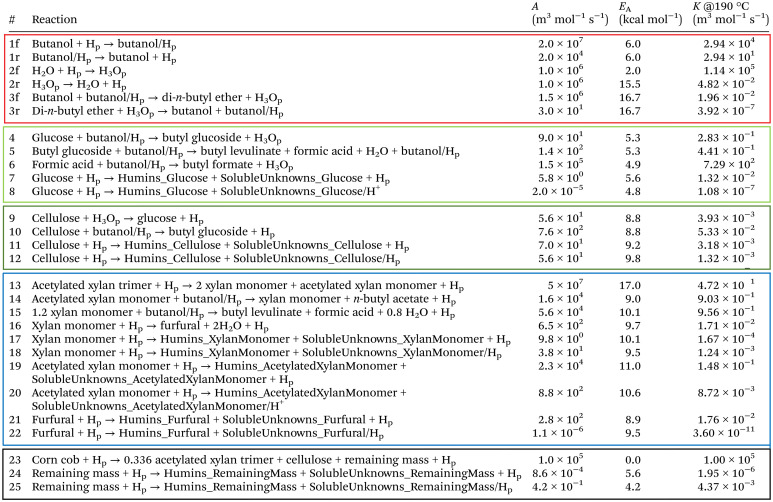

A notable operational difference was observed in mixture viscosity. While ethanolysis tolerates lignocellulosic biomass loadings of up to 20 wt% without impairing analysis,^22,23^ preliminary butanolysis trials at the same loading produced a highly viscous product mixture unsuitable for reliable gas chromatography quantification. All subsequent experiments were therefore performed at 10 wt% feedstock loading to ensure analytical accuracy. This higher viscosity is likely related to polymerisation reactions, potentially promoted by the furfural concentrations observed in butanolysis, although confirming this effect lies beyond the scope of the present study.

##### 3.3.3.2. n-Butanol and di-n-butyl ether

In the surrogate kinetic model, *n*-butanol is both the solvent and a reactant, with its behaviour modelled following the ethanolysis framework of O'Shea *et al.*^23^ but adapted for the distinct chemical environment of *n*-butanol. The model incorporates the reversible, acid-catalysed etherification of *n*-butanol to di-*n*-butyl ether, represented as:11
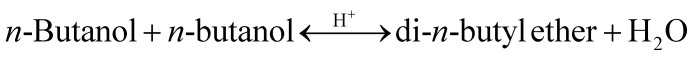


This step is modelled alongside the protonation and deprotonation equilibria of *n*-butanol and water, informed by density functional theory (DFT) calculations.^60^ Sulphuric acid is assumed to fully dissociate in *n*-butanol, providing two protons (H^+^) to catalyse both etherification and saccharide conversion pathways. Inclusion of this competitive pathway is essential, as ether formation consumes *n*-butanol that would otherwise participate in the formation of *n*-butyl levulinate.

By incorporating the etherification and protonation equilibria directly into the surrogate kinetic model, the *n*-butanol reaction network is represented in a way that is consistent with the hierarchical framework established for ethanolysis. This ensures that the competitive consumption of *n*-butanol through ether formation, as well as its role in saccharide conversion to *n*-butyl levulinate, is captured without altering the underlying structure of the model. In this way, the butanolysis adaptation preserves mechanistic consistency with ethanolysis while accurately reflecting the distinct reactivity of *n*-butanol.

##### 3.3.3.3. n-Butyl levulinate

*n*-Butyl levulinate formation is modelled in both the glucose and hemicellulose (xylan) submodels of the surrogate kinetic framework. Insights from ethanolysis studies^23^ show that glucose is first converted to ethyl glucoside, which then undergoes acid-catalysed rearrangement and hydration to form ethyl levulinate. In the butanolysis model, a similar acid-catalysed pathway is assumed for glucose, proceeding *via n*-butyl glucoside, and for cellulose through sequential depolymerisation to glucose followed by butyl levulinate formation.

Due to limited mechanistic data for xylan conversion to butyl levulinate, its pathway is represented by a stoichiometrically balanced reaction analogous to cellulose but adjusted for the C_5_ sugar composition. The three global reactions are:12
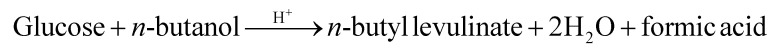
13
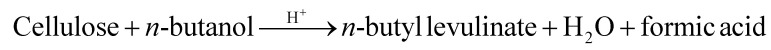
14
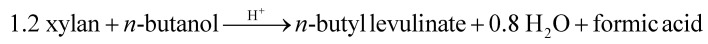


These lumped reactions capture the net stoichiometry of butyl levulinate formation from each carbohydrate source, enabling consistent integration into the hierarchical kinetic model (Fig. 4 and Table 2) while preserving mass balance and mechanistic plausibility.

##### 3.3.3.4. n-Butyl formate

During acid-catalysed butanolysis, *n*-butyl levulinate formation from both cellulose and hemicellulose generates water and formic acid as by-products. Within the surrogate kinetic model, formic acid is further consumed *via* esterification with *n*-butanol to yield *n*-butyl formate and water:15
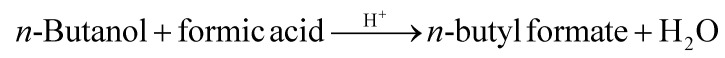


This pathway is supported by the well-established acid-catalysed esterification of carboxylic acids with alcohols, with analogous formation of ethyl formate from ethanol and formic acid reported under similar conditions.^61^ The reaction is expected to proceed efficiently in the butanol-rich medium, consistent with the experimentally observed *n*-butyl formate concentrations. Incorporating this step improves the mass balance for formic acid in the reaction network and enhances the model's ability to capture secondary product formation, thereby increasing predictive accuracy and providing a mechanistic basis for future process optimisation.

##### 3.3.3.5. n-Butyl acetate

Acid-catalysed butanolysis of acetylated hemicellulose produces *n*-butyl acetate *via* cleavage of acetyl substituents and subsequent esterification with *n*-butanol:16
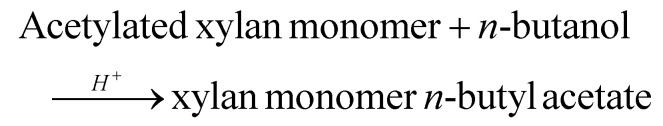


Significant formation of *n*-butyl acetate was observed for xylan and corn cob, reaching up to 41 wt% relative to *n*-butyl levulinate, whereas glucose and cellulose yielded only trace amounts. A control experiment using xylopyranose, which lacks acetyl groups, produced negligible *n*-butyl acetate, confirming its origin in acetyl substituents of hemicellulosic fractions.

^1^H NMR spectroscopy measurements of the isolated xylan from corn core revealed a monomer-based degree of acetylation of 0.34. This corresponds to approximately one acetyl group per three anhydrous xylopyranose units, assuming no other substituents are present. Similar quantities of *n*-butyl acetate were observed in reactions with both isolated xylan and corn cob, implying either a higher degree of acetylation in the native xylan within corn cob or the presence of additional acetylated species in the biomass matrix, such as lignin.

The proposed reaction mechanism involves the acid-catalysed cleavage of acetyl groups from the xylan backbone, which then react with protonated *n*-butanol to yield *n*-butyl acetate and regenerate H^+^. This esterification pathway is analogous to mechanisms proposed in hydrolysis systems for acetic acid and aligns with observed reaction behaviour in butanolysis systems.^62–66^

Following deacetylation, the liberated xylopyranose monomer may undergo further transformation to products such as *n*-butyl levulinate or furfural and contribute to humin formation.^67,68^ Incorporation of this acetyl-driven esterification mechanism into kinetic models significantly improves their predictive capability concerning product distributions and better reflects the underlying chemistry of lignocellulosic butanolysis.

##### 3.3.3.6. Furfural

Furfural was detected as a transient intermediate in the butanolysis of xylan and corn cob but was absent in glucose and cellulose reactions. This is consistent with its formation *via* acid-catalysed dehydration of xylose, the principal hemicellulosic sugar released during xylan depolymerisation, as previously reported.^63,69,70^17
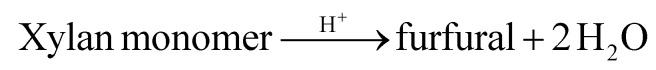


This pathway, adapted from established hydrolysis chemistry, has been incorporated into the kinetic model, extending earlier ethanolysis-based frameworks.^22,23^ Although furfural is not proposed as an intermediate in *n*-butyl levulinate formation, it is a key precursor to humins. To investigate this role, furfural (10 wt%) was subjected to butanolysis conditions (1 wt% H_2_SO_4_, reaction times and temperatures as herein) and yielded predominantly insoluble solids with negligible supernatant, indicating its rapid polymerisation under acidic, butanol-rich conditions. While the detailed mechanistic contribution of furfural to humin formation remains outside the scope of this study, its explicit inclusion in the reaction network is essential for accurate kinetic modelling and for guiding future strategies to suppress humin formation.

##### 3.3.3.7. Water

In the acid-catalysed butanolysis of lignocellulosic biomass, water is generated as a stoichiometric by-product of key transformation steps, including carbohydrate esterification to *n*-butyl levulinate and etherification of *n*-butanol. Within the surrogate kinetic model, water production is determined directly from the balanced reaction scheme (Table 2), ensuring internal mass conservation across all sub-models. Accurate quantification of water is essential, as it influences reaction equilibria, proton activity, and potential hydrolysis side reactions. The validity of this stoichiometric treatment is supported by near-complete mass closure in glucose butanolysis experiments, where over 99% of the initial carbon mass was accounted for in measured products and humins. Incorporating water explicitly into the hierarchical reaction mechanism ensures mechanistic fidelity and strengthens predictive capability for process optimisation.

##### 3.3.3.8. Humins

Humins, defined here as the insoluble solid residues generated during acid-catalysed butanolysis, are quantified experimentally by centrifuging the reaction mixture, recovering the solids, and drying them to constant mass. Elemental composition (CHNOS) is subsequently determined *via* elemental analysis, and the resulting data are incorporated into the surrogate kinetic model to ensure accurate elemental mass closure.

Humins are formed through complex polymerisation and condensation reactions, primarily involving dehydration products of carbohydrates such as 5-hydroxymethylfurfural (HMF) and furfural, along with phenolic fragments derived from lignin-rich fractions.^71,72^ In the butanolysis system, the “remaining mass” sub-model captures these transformations, with humin formation modelled as the sole fate of non-carbohydrate solids, in addition to contributions from carbohydrate-derived intermediates. Although humins are traditionally regarded as an undesirable sink of carbon that reduces liquid fuel yields, emerging studies highlight their potential applications in materials, carbon sequestration, and soil amendment.^73^

Including humins explicitly in the reaction network is therefore essential, both to accurately represent carbon flows within the system and to inform process design strategies that either minimise their formation to maximise fuel yields or valorise them as co-products in an integrated biorefinery context.

##### 3.3.3.9. Soluble unknowns

For corn cob butanolysis, overall mass balances were close at approximately 92%, indicating that a fraction of the reaction products was not directly identified. To maintain mass conservation in the kinetic model, these unquantified soluble species were grouped into a composite category termed “soluble unknowns”. Prior to analysis, all solid residues were removed by centrifugation and weighed, ensuring that the remaining unaccounted mass could be attributed exclusively to dissolved, unidentified products in the liquid phase.

In the reaction network, soluble unknowns are generated *via* mass-conservation pathways from multiple sources, including the degradation of cellulose, glucose, xylan, acetylated xylan, furfural, and residual biomass. While this fraction is likely composed of a diverse range of low-concentration intermediates and secondary products, its overall contribution to the total product spectrum remains small, as all major liquid and solid products have been explicitly quantified. Nevertheless, calculating the inferred elemental composition of soluble unknowns offers valuable clues to their molecular characteristics, possible formation pathways, and roles within the reaction network. Incorporating this fraction into the surrogate kinetic model not only enforces elemental mass closure but also ensures that minor species contributions are reflected in process-scale yield predictions.

#### 3.3.4. Chemical reaction kinetic theory, modelling and optimisation

The kinetic modelling in this work utilises Cantera,^60^ an open-source mathematical solver for problems involving chemical kinetics and thermodynamics. A homogeneous 0-D reactor model is used, where all state variables are functions of time and thermodynamic equilibrium. The initial thermodynamic and chemical state is defined by the declaration of pressure, temperature, and species mass fraction, with the ideal gas equation of state employed.

The heating and isothermal phases of the reactor are modelled using a reactor network consisting of a reactor interconnected through a heat-conducting wall with an external reservoir at the given reaction. At *t* = 0, the reactor is at 25 °C. The heat flux, *q*, through the wall is computed by *q* = *U*(*T*_reactor_ − *T*_reservoir_), where *U* is the heat transfer coefficient, and *T*_reactor_ and *T*_reservoir_ are the temperatures of the reactor and reservoir, respectively. *U* is optimised so that the temperature profile of the model reactor maps its experimental temperature profile in the aluminium block.

A set of ordinary differential equations is numerically solved using Cantera to describe the concentrations of the species in the system as a function of time. An exemplar differential conversion equation for glucose in ethanolysis reaction is given by eqn (18):18
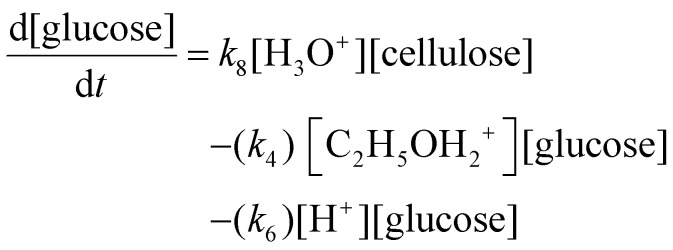
where *k*_8_ is the rate constant for the production of glucose from cellulose, and *k*_4_ and *k*_6_ are the rate constants for the conversion of glucose to ethyl glucoside and “unknowns_Glucose_”, respectively. All reactions progress according to their rate constant, *k*, with Arrhenius temperature dependence as shown in eqn (19):19
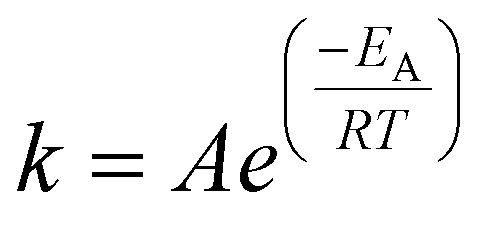
Each *k* is defined by an activation energy (*E*_A_) and a pre-exponential coefficient (*A*). Each *E*_A_ and *A* are determined by a hierarchical, sub-model by sub-model optimisation procedure, where computed and experiment-derived time-resolved species fractions are compared.

The optimisation procedure was performed using Phase 1 of the Machine Learned Optimisation of Chemical Kinetics (MLOCK).^74^ MLOCK generates an extensive library of kinetic model candidates (80 000) where the rate constant parameters, *E*_A_ and *A*, of each reaction is randomly assigned across a *p* range up to the diffusion limit, 1 × 10^−13^ cm^3^ mol^−1^ s^−1^. MLOCK then computes the model at each condition for which experimental data is available, and the error between the model candidate species fractions and those of experiment is calculated according to the least square error averaged across each experimental condition:20
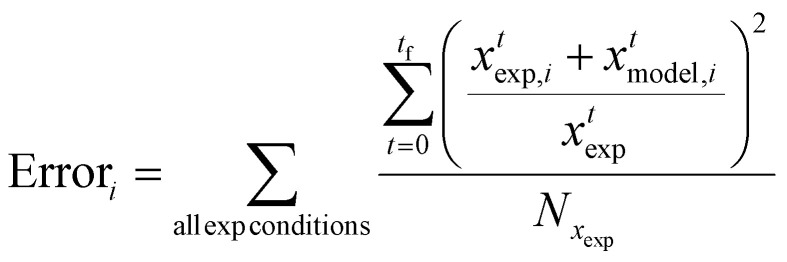
where *x*^*t*^_exp,*i*_ is the mass of species *i* measured experimentally after time *t*, *x*^*t*^_model,*i*_ is the mass of species *i* calculated by the model after time *t*, and *N*_*x*_exp__ is the number of experimental data points at that condition. The errors observed are used to set narrower ranges of *E*_A_ and *A* for each reaction through a bound-refinement algorithm. A second library of 80 000 model candidates is generated at these refined ranges. A genetic seed scan is then performed centred on the resulting model candidate with the lowest error. The model candidate exhibiting the lower error function after these three iterations of optimisation is deemed to be the optimised model, and the corresponding rate constants are selected as the empirical rate constants, which allows for the best reproduction of the experimental data.

Each sub-model in Table 2 and Fig. 4 is optimised individually in hierarchical order to the corresponding experimental data based on the molecular complexity of the feedstock. Once derived, the rate constants for each sub-model are fixed in the overall system during subsequent optimisations.

## 4. Results and discussion

### 4.1. Feedstock characterisation

#### 4.1.1. Elemental analysis of feedstocks

The ultimate analysis of glucose, cellulose, corn cob, and wheat straw highlights key elemental compositions that influence their suitability as feedstocks for alcoholysis to produce *n*-butyl levulinate (Table 3). The carbon content, ranging from 40.0% in glucose to 45.0% in corn cob, is a critical factor in determining the theoretical yield of carbon-based products such as alkyl levulinates. The hydrogen-to-carbon (H/C) molar ratio, indicative of feedstock reactivity and fuel potential, decreases progressively from glucose (2.00) to corn cob (1.72), reflecting increasing molecular complexity, and the recalcitrant nature of lignocellulosic materials. The low nitrogen content in all feedstocks minimises nitrogenous by-products, reducing the need for post-synthesis removal. In addition, nitrogen-rich biomass may require more acid for effective hydrolysis due to buffering effects and may also alter the cell wall structure of real biomass, affecting the accessibility of cellulose and hemicellulose by acids. This elemental variability, particularly between glucose (simple sugar) and wheat straw or corn cob (lignocellulosic biomass), underscores the importance of optimising reaction conditions to accommodate feedstock-specific characteristics in alcoholysis processes.

**Table 3:** Ultimate analysis (wt%) of dried feedstocks

Feedstock	Carbon	Hydrogen	Nitrogen	H/C molar ratio
Glucose	40.0	6.7	0.0	2.00
Cellulose	42.0	6.1	0.0	1.73
Xylan	41.7	6.0	0.0	1.84
Corn cob	45.0	6.5	1.1	1.72

#### 4.1.2. Biochemical analysis of corn cob and wheat straw

Both wheat straw and corn cob, belonging to the Poaceae family and classified under the genera *Triticum* and *Zea*, respectively, are characterised by their high cellulose and hemicellulose contents, which are advantageous for alcoholysis processes. The biochemical compositions reveal that wheat straw contains 32.1% glucan and 26.9% hemicellulose, whereas corn cob contains 29.7% glucan and 26.9% hemicellulose, indicating comparable carbohydrate availability. These compositions make both substrates suitable for producing alkyl levulinates. Additionally, the relatively lower lignin content in corn cob (11.7%) compared to wheat straw (16.7%) suggests it may offer less mechanical resistance to decomposition during alcoholysis, potentially facilitating more efficient conversion to target products such as *n*-butyl levulinate. The lignin content significantly affects the acid hydrolysis of lignocellulosic biomass because it is a complex, hydrophobic polymer that encases cellulose and hemicellulose, making it harder for acid to access and hydrolyse the polysaccharides. In addition, a high lignin content might decrease the acid concentration by degrading itself into phenolic compounds.^75–78^ Such compositional insights emphasise the critical role of feedstock variability in optimising alcoholysis for biofuel production.

The presence of extractives in lignocellulosic biomass can significantly influence the acid-catalysed butanolysis process. These non-structural components, such as phenolics, fats, waxes, and low-molecular-weight sugars, may interfere with the hydrolysis of cellulose and hemicellulose by reacting with degradation products or consuming the acid catalyst. As a result, they can promote undesired side reactions such as humin formation and the generation of inhibitory compounds, ultimately reducing the yields of *n*-butyl levulinate.^79^ Consequently, the removal of extractives through preliminary solvent extraction can be an alternative to enhance *n*-butyl levulinate yield, minimise acid consumption, and improve the overall efficiency and reproducibility of sulphuric acid-based pretreatments. However, it must be highlighted that for the sake of simplicity and from a sustainable point of view, the use of raw biomass without previous treatment is desired because the extractives removal often employs a large amount of organic solvents (mainly derived from non-renewable sources) and thus generates a lot of chemical waste.^80^ Thus, no pretreatment of the biomass is employed for the processes used in this study.

The inorganic fraction of lignocellulosic biomass, commonly referred to as ash, plays a critical role in acid-catalysed butanolysis processes. Alkali and alkaline earth metals, such as K, Na, Ca, and Mg, can partially neutralize sulphuric acid, decreasing the effective acidity of the reaction medium. This reduction in proton availability impairs the hydrolysis of structural carbohydrates and can diminish the formation of target *n*-butyl levulinate. Additionally, metal ions from ash may catalyse side reactions or facilitate the precipitation of humins, affecting both the product yields and process viability.

**Fig. 5:**
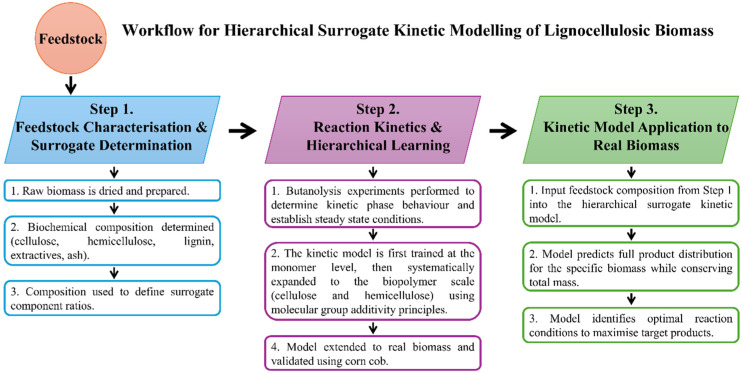
Three-step hierarchical surrogate kinetic modelling workflow, comprising feedstock characterisation, hierarchical development of monomer to biopolymer kinetic models, and application of the model to real biomass to predict product distributions and identify optimal reaction conditions.

The biochemical composition of the target lignocellulosic biomass is relevant to surrogate chemical kinetic models, which are tailored to predict the formation of key species in the reaction mixture, including *n*-butyl levulinate, *n*-butanol, di-*n*-butyl ether, water, humins, furfural, *n*-butyl formate, and *n*-butyl acetate. These models can be trained against experimental data to simulate reaction kinetics and steady-state yields under varying conditions by incorporating the cellulose, hemicellulose, lignin, and other constituent fractions. This adaptability is especially advantageous given the inherent variability in biomass composition, which fluctuates with species, growth season, and climatic conditions. Such predictive models enable a robust and scalable approach to optimising alcoholysis for diverse feedstocks, ensuring high efficiency and economic feasibility in biofuel production. This three-step workflow, from feedstock characterisation through hierarchical kinetic model development to model application, is summarised in Fig. 5.

#### 4.1.3. Degree of acetylation of xylan from corn core

The concentration of acetyl groups in xylan was determined using ^1^H NMR analysis and two assumptions. First, that each xylopyranose monomer was considered to bear either zero or one acetyl group (monoacetylation assumption). This simplifies the analysis by treating all acetylated monomers as having a single acetyl group, while unacetylated monomers retain both hydroxyl groups. While this assumption may not fully capture the exact molecular distribution, since some monomers could bear more than one acetyl group, it provides an accurate estimate of the average degree of acetylation, which is sufficient for reliably predicting product yields in butanolysis reactions.

**Fig. 6:**
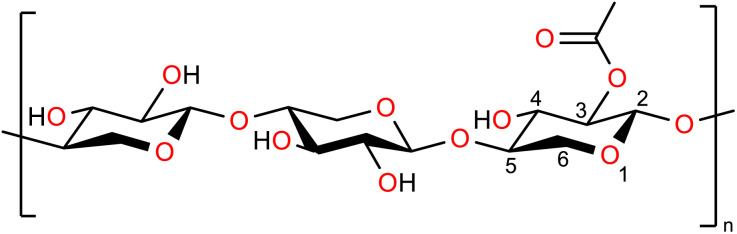
Molecular structure of three linked xylan monomers, two unacetylated (left and central units) and one acetylated at position 3 (right unit).

Second, it was assumed that acetyl groups account for all substituents present in xylan. This is supported by the absence of peaks corresponding to other substituents in the NMR spectra, with all signals attributable to saccharides or acetyl groups. Minor saccharide side chains, such as arabinose, are considered functionally equivalent to additional xylose units for this analysis. Duplicate analyses yielded highly consistent results, with a mean monomer-based degree of acetylation of 0.34 as shown in Fig. 6. These findings confirm that acetyl substituents are a significant component of the xylan structure, supporting the hypothesis that *n*-butyl acetate formation during butanolysis originates from acid-catalysed cleavage of these acetyl groups. The comparable levels of *n*-butyl acetate observed in reactions with both isolated xylan and corn cob further suggest that xylan-bound acetyl groups are the primary contributors to *n*-butyl acetate formation in lignocellulosic biomass.

### 4.2. Steady-state yields of *n*-butyl levulinate

**Fig. 7:**
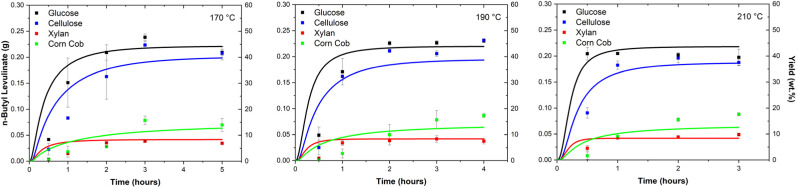
*n*-Butyl levulinate (g) produced by acid-catalysed butanolysis of glucose, cellulose, xylan, and corn cob at 170, 190, and 210 °C for various reaction times. The square symbols represent experimental data points, while the lines were produced by the surrogate kinetic model. The error bars represent the standard deviation of the four data points collected for each set of experimental conditions.

Fig. 7 shows the evolution of *n*-butyl levulinate concentration from the four feedstocks investigated, over reaction times of 0.5–5 h at 170, 190, and 210 °C. The corresponding maximum yields obtained under these conditions are summarised in Table 5. Specifically, Table 5 reports the highest mass, molar, and carbon yields achieved for the conversion of each feedstock (10 wt%) to *n*-butyl levulinate in *n*-butanol (89 wt%) using sulphuric acid (1 wt%) as catalyst.

#### 4.2.1. *n*-Butyl levulinate mass yields

Mass yields of *n*-butyl levulinate were highest for glucose and cellulose across all conditions, with optimal values observed at temperatures ranging from 170 to 190 °C. Glucose achieved a maximum mass yield of (47.4 ± 2.7)% at 170 °C, followed by a slight decline to (45.9 ± 1.1)% at 190 °C and further to (40.5 ± 0.9)% at 210 °C. Similarly, cellulose showed comparable performance, peaking at (46.2 ± 0.7)% at 190 °C before decreasing to (39.0 ± 4.8)% at 210 °C. As glucose and cellulose are simpler structures, the increase in the reaction temperature favours the formation of additional side products, such as humins.

For corn cob, the maximum mass yield of (17.5 ± 3.1)% was attained at 210 °C. In a novel finding, *n*-butyl levulinate was also formed from xylan, demonstrating that levulinate formation occurs from both the cellulose and hemicellulose constituents of the biomass. Although xylan achieved comparatively lower yields of *n*-butyl levulinate: (9.7 ± 3.7)% at 210 °C, hemicellulose must now also be included in molar yield calculations. The more complex feedstocks, xylan and corn cob, achieved their highest yields at 210 °C, underscoring the recalcitrance of lignocellulose and demonstrating that higher temperatures promote the cleavage of carbohydrates and sugars from the lignocellulosic matrix. As mentioned, the presence of lignin in the corn cob and wheat straw (Table 4) represents the recalcitrance in the acid-catalysed butanolysis, indicating that higher temperatures are required for the H^+^ to penetrate inside the plant cell. However, higher temperatures also favour the formation of di-*n*-butyl ether and other side reactions (many of which result from dehydration reactions), posing a trade-off that may hinder the commercial feasibility of one-pot alcoholysis processes.

**Table 4:** Biochemical analysis (wt%) of wheat straw and corn cob

Feedstock	Cellulose	Hemicellulose					Lignin		Extractives	Ash	Unknowns
	Glucan	Xylan	Arabinan	Galactan	Mannan	Rhamnan	Klason lignin	Acid-soluble lignin			
Wheat straw	32.1	26.9					16.7		19.2	4.3	0.8
		22.9	2.6	0.8	0.4	0.2	14.7	2.0			
Corn cob	29.7	26.9					11.7		23.4	3.1	5.2
		24.2	2.1	0.5	0.1	0	8.6	3.1			

#### 4.2.2. *n*-Butyl levulinate molar yields

Molar yields followed a similar pattern to mass yields. Glucose reached a peak molar yield of (49.6 ± 2.7)% at 170 °C, whereas cellulose peaked at (43.4 ± 0.7)% at 190 °C. For corn cob and xylan, the highest molar yields (28.8 ± 3.1) and (8.9 ± 3.7)%, respectively, were observed at 210 °C. For corn cob, this peak reflects the molecular efficiency of the process. Conversely, the discrepancy between mass-based and mole-based yields is because molar yield considers only the fractions of the biomass (cellulose and hemicellulose) that directly contribute to *n*-butyl levulinate production, as mentioned in Section 3.

Previous investigations on hydrolysis, ethanolysis, and butanolysis, report molar yield considering only the cellulose contribution to *n*-butyl levulinate,^25,26,81^ artificially inflating the yield and obscuring the actual molecular efficiency of such processes. This work proposes that molar yield should always be reported in accordance with the methods outlined in Section 3.

#### 4.2.3. *n*-Butyl levulinate carbon yields

Carbon yields, which measure the proportion of biomass-derived carbon contributing to *n*-butyl levulinate formation, showed trends analogous to the mass and molar yields. Glucose and cellulose achieved the highest carbon yields of 41.3% and 38.3% at 170 °C and 190 °C, respectively. Corn cob achieved a maximum carbon yield of 14.1% at 210 °C, while xylan reached its optimal carbon conversion of 8.9% at the same temperature.

These results demonstrate that, under optimised conditions, a substantial proportion of feedstock carbon can be successfully directed toward *n*-butyl levulinate production, particularly from glucose and cellulose. However, lower carbon yields to *n*-butyl levulinate indicate significant carbon losses to side products or insoluble residues for lignocellulosic substrates such as corn cob and xylan. To better account for the fate of feedstock carbon and assess overall process efficiency, the carbon molar yields of the humins formed are reported and discussed in Section 4.5.2.

#### 4.2.4. Temperature effect and feedstock-specific observations

Optimal yields for glucose and cellulose occurred at 170–190 °C, reflecting their simpler structures and lower energy requirements for depolymerisation and conversion. However, yields for these feedstocks declined at 210 °C, likely due to decomposition of the product or the formation of other liquid by-products. In contrast, corn cob and xylan required higher temperatures (190–210 °C) to reach their peak yields. For corn cob, this is attributed to its more complex chemical composition and recalcitrance, particularly associated with the lignin fraction bound within the lignocellulosic matrix, demanding more thermal energy to break down into reactive intermediates.

Despite the superior performance of higher-temperature configurations in producing *n*-butyl levulinate from lignocellulosic biomass, more moderate reaction temperatures offer a clear advantage in minimising side reactions. One significant side reaction is the formation of di-*n*-butyl ether, which not only consumes the costly alcohol (a costly reagent which, on average, accounts for 38% of the cost of the process^4^) but also generates water, complicating downstream processing. Suppressing this undesired reaction is therefore a key objective of this study and can be achieved by reducing both the acid concentration and the reaction temperature.

Among all the temperatures tested, 190 °C emerged as the optimal condition, striking a balance between achieving high *n*-butyl levulinate yields and limiting ether formation. This temperature facilitated effective conversion across a range of biomass feedstocks of different complexity, including more recalcitrant materials, while mitigating the extent of etherification. As such, 190 °C represents a compromise that enhances overall process selectivity and economic efficiency.

#### 4.2.5. Steady-state conditions

In this study, the steady-state reaction time is defined as the shortest duration at which the mass of *n*-butyl levulinate remains constant (within ±10% experimental uncertainty) over three consecutive hourly samples. As reported in Section 4.2.4, 190 °C was identified as the optimum temperature for maximising *n*-butyl levulinate yields across all four feedstocks examined. At this temperature, glucose, cellulose, and xylan attained a steady-state within 2 hours, whereas the more recalcitrant corn cob feedstock required 3 hours.

Steady-state times lengthen as feedstock complexity increases (glucose < cellulose < xylan < corn cob) and increase further at lower temperatures owing to slower kinetics. Notably, the side-reaction forming di-*n*-butyl ether does not reach equilibrium under any of the conditions tested. Therefore, reaction durations should be carefully limited to minimise ether formation. While maximum *n*-butyl levulinate yields do not always coincide with the earliest steady-state time points, the highest levulinate-to-ether ratios are consistently observed at these earlier stages. This finding underscores the importance of accurately defining steady-state conditions when performing techno-economic and life-cycle assessments for potential commercial applications. Overall, the results highlight the critical interplay between feedstock reactivity, temperature, and reaction time in optimising process efficiency and product selectivity.

### 4.3. The ether effect

The formation of di-*n*-butyl ether is a significant side reaction in acid-catalysed butanolysis, arising from the dehydration of two *n*-butanol molecules in the presence of an acid catalyst, as described by eqn (11). The phenomenon parallels diethyl ether production in ethanolysis reactions and can substantially influence process optimisation and techno-economic viability as it consumes the costly alcohol and produces water. In this way, a derivative molecule is generated, making the entire process less environmentally sustainable. Although it has been shown that blends of the alkyl levulinate, alcohol, and dialkyl ether can enhance fuel properties relative to any individual component,^19,20^ these mixtures often contain comparatively small concentrations of the dialkyl ether. Thus, its formation should be minimised for commercial processes.

**Fig. 8:**
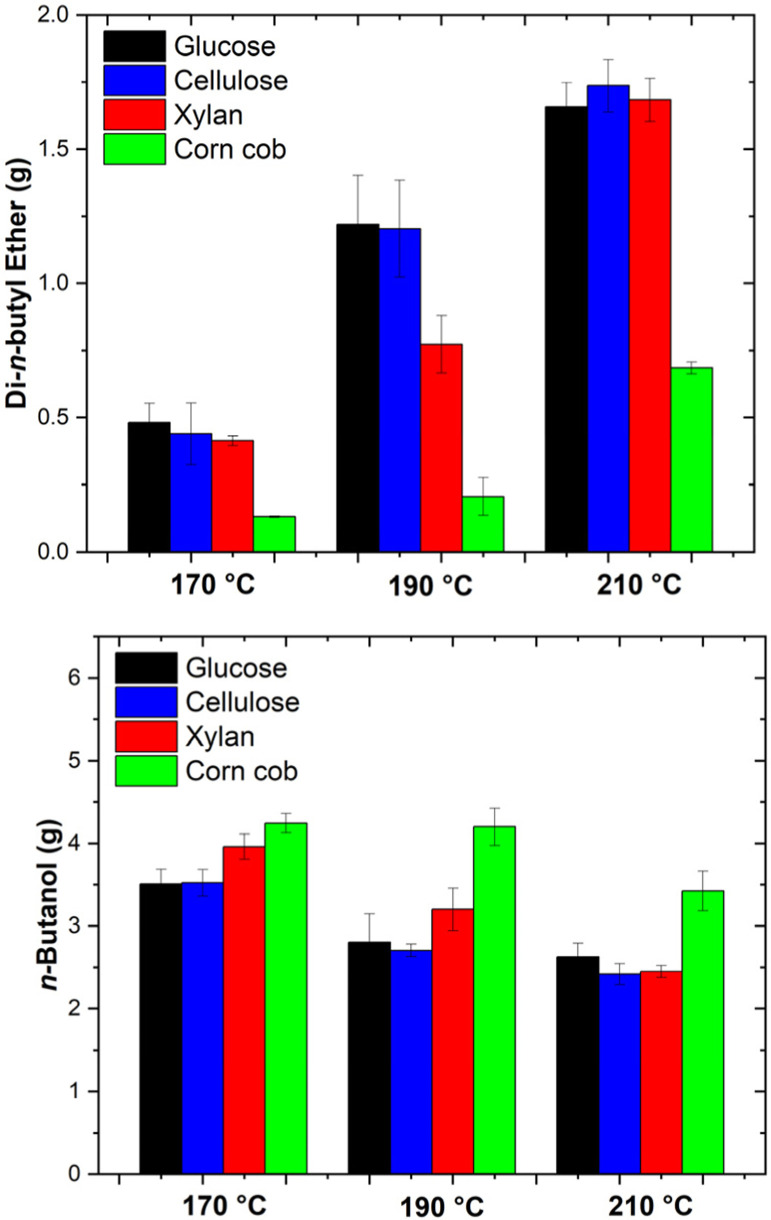
Mass of di-*n*-butyl ether (g) and *n*-butanol (g) produced *via* the sulphuric acid-catalysed butanolysis of glucose, cellulose, xylan, corn cob, and wheat straw at 170, 190, and 210 °C for a reaction time of 2 h. The error bars represent the standard deviation of the four data points collected for each set of experimental conditions.

Fig. 8 shows the concentrations of di-*n*-butyl ether produced from the feedstocks at two hours over the three temperatures. In many studies, efforts focus on maximising *n*-butyl levulinate yield, with comparatively little attention paid to the accumulation of di-*n*-butyl ether.^40^ Although high di-*n*-butyl ether concentrations can increase downstream separation costs, reducing *n*-butanol availability for *n*-butyl levulinate production and diminishing overall process efficiency, this variable has been frequently overlooked.^26^ Consequently, we have integrated this quantity into our analysis, which involves measuring di-*n*-butyl ether concentrations over time using gas chromatography (GC) and confirming the results *via* mass spectrometry (MS). This approach reveals that di-*n*-butyl ether formation tends to accelerate under conditions of elevated temperature, higher acid loading, and extended reaction times.

In terms of techno-economics, targeting the maximum *n*-butyl levulinate yield alone may be suboptimal. Instead, reaction severity, encompassing temperature, acid concentration, and reaction duration, must be carefully balanced to moderate di-*n*-butyl ether formation while still achieving commercially relevant *n*-butyl levulinate outputs. A detailed understanding of the relative rates of *n*-butyl levulinate and di-*n*-butyl ether formation is crucial to devising efficient, cost-effective butanolysis processes at an industrial scale. Thus, the optimal reaction times achieved are the shortest at which steady-state of *n*-butyl levulinate production is observed and are reported in Table 5.

**Table 5:** *n*-Butyl levulinate maximum yields, reaction conditions, and steady-state reaction times

Feedstock	Temp. (°C)	Steady-state time (h)	*n*-Butyl levulinate yields		
			wt %	Molar %	Carbon %
Glucose	170	2	47.4	49.6	41.3
	190	2	45.9	48.0	40.0
	210	0.5	40.5	42.5	35.3
Cellulose	170	3	44.4	41.8	36.9
	190	2	46.2	43.4	38.3
	210	1	39.0	36.7	32.4
Xylan	170	2	7.7	7.0	6.5
	190	2	8.2	7.6	7.0
	210	1	9.7	8.9	8.3
Corn cob	170	3	15.7	25.8	12.6
	190	3	17.2	28.4	13.9
	210	2	17.5	28.8	14.1

### 4.4. Major soluble species fractions

**Fig. 9:**
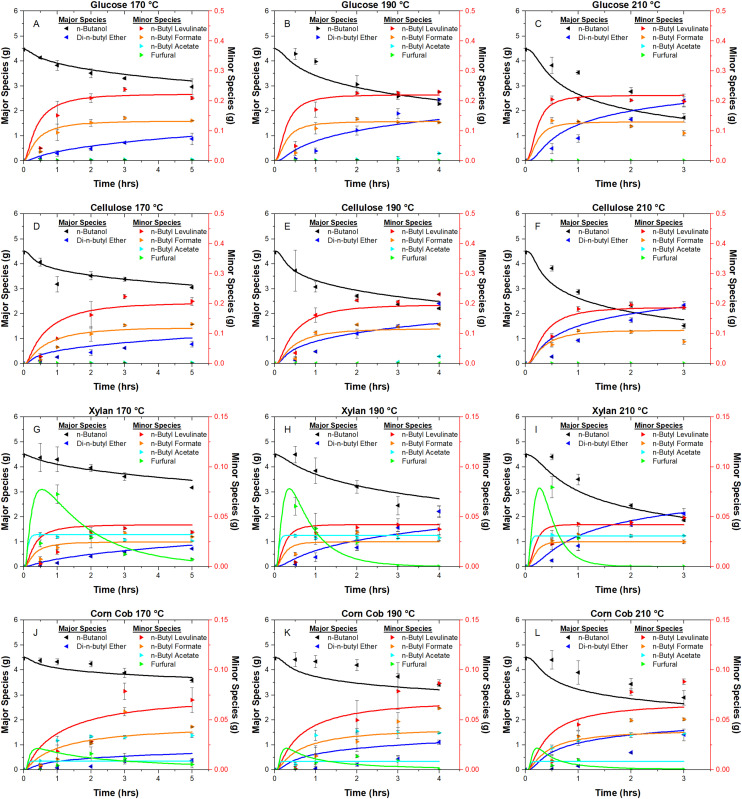
Liquid product concentrations (g) for the acid-catalysed butanolysis of glucose (A–C), cellulose (D–F), xylan (G–I), and corn cob (J–L) at 170, 190, and 210 °C. The square symbols represent experimental data points, while the lines were produced by the surrogate kinetic model. The error bars represent the standard deviation of the four data points collected for each set of experimental conditions.

Fig. 9 presents the concentrations of both major and minor species detected during the sulphuric acid-catalysed butanolysis of glucose, cellulose, xylan, and corn cob. All products were quantified using gas chromatography and their identities confirmed by mass spectrometry techniques.

#### 4.4.1. *n*-Butanol and di-*n*-butyl ether

The primary compounds detected in all feedstock reactions were *n*-butanol and di-*n*-butyl ether, with the latter (formed as described by reactions 3f and 3r in Table 2) emerging as the most abundant by-product for most conditions. Because both compounds are present in significant quantities, they dominate the overall mass balance for the mixtures.

#### 4.4.2. *n*-Butyl levulinate

*n*-Butyl levulinate was observed in all reaction mixtures, confirming that levulinates can form from a wide range of biomass-derived substrates. To the authors’ knowledge, this is the first study reporting the formation of *n*-butyl levulinate from xylan using a one-pot process. Notably, this unveils that levulinates can be produced from hemicellulose at relatively high yields (9.7 wt% or 8.9 mol%). As discussed in Section 3, all molar yields in this paper were calculated based on the sum of monomeric units in both the cellulose and hemicellulose fractions of the biomass, accounting for the newly confirmed pathway (as described in eqn (3) and (4)).

#### 4.4.3. *n*-Butyl formate, *n*-butyl acetate & furfural

Several co-products were consistently or selectively observed during the acid-catalysed butanolysis of biomass-derived substrates, reflecting the complex interplay of primary and secondary reaction pathways under thermochemical conditions.

*n*-Butyl formate was detected in all reaction mixtures at concentrations slightly lower than *n*-butyl levulinate. Its formation is attributed to the esterification of *n*-butanol with formic acid, a known degradation by-product of C6 sugar pathways associated with levulinate formation (see reaction 6, Table 2). Its consistent presence underscores the unavoidable coupling of main and side reactions in this system.

In contrast, *n*-butyl acetate was formed in appreciable amounts only in reactions involving xylan and corn cob, where it reached steady-state concentrations up to 41 wt% relative to *n*-butyl levulinate (Fig. 9). Only trace levels were observed in glucose, cellulose, and xylose systems.

This selective formation implies a mechanism involving direct transesterification of acetyl groups from acetylated hemicellulose components with *n*-butanol. ^1^H NMR analysis supports this, indicating a degree of acetylation of 0.34 for the xylan used (Section 4.1.3). Comparable yields in corn cob suggest either a higher intrinsic acetylation of embedded xylan or the contribution of acetyl groups from other constituents, such as lignin or starch-like polysaccharides.

Furfural, a well-known dehydration product of pentoses, was detected only in xylan and corn cob reactions, consistent with its origin from xylan degradation. It typically appeared at early time points and diminished over time due to further transformation into humins and other non-volatile products. A complementary experiment using furfural as the sole feedstock resulted in rapid polymerisation, precluding GC analysis, confirming its high reactivity under these conditions.

These observations collectively highlight the influence of feedstock composition on the distribution of both major and minor products. The overlapping formation pathways further emphasise the mechanistic complexity of alcoholysis reactions and the need to consider side reactions when optimising yield and selectivity.

### 4.5. Humins

#### 4.5.1. Humin composition

The elemental compositions presented in Table 6 illustrate a consistent trend across all feedstocks: the humins formed during butanolysis exhibit elevated carbon content and reduced hydrogen-to-carbon (H/C) molar ratios relative to their biomass source. This shift in composition reflects the increasing aromatic character and condensed structure, typical of humin formation *via* dehydration and polymerisation reactions under acidic conditions. The substantial decrease in H/C ratio from 1.84 in xylan to 1.12 in xylan-derived humins, for instance, is indicative of greater unsaturation and aromaticity, hallmarks of thermally stable carbonaceous residues.

**Table 6:** Ultimate analysis (mol%) of feedstocks and corresponding humins formed during butanolysis

Sample	C	H	N	S	H/C
Glucose	3.75	6.44	0.00	0.00	1.72
Glucose humins	5.38	5.55	0.00	0.04	1.03
Cellulose	3.55	6.31	0.00	0.00	1.78
Cellulose humins	4.95	5.78	0.00	0.20	1.17
Xylan	3.47	6.38	0.00	0.00	1.84
Xylan humins	5.19	5.83	0.00	0.08	1.12
Corn cob	3.75	6.44	0.08	0.00	1.72
Corn cob humins	4.03	5.79	0.09	0.13	1.44

Notably, none of the feedstocks contain detectable levels of sulphur, yet the humins contain varying amounts, ranging from 0.04 to 0.20 mol%. This incorporation arises from the use of a sulphuric acid catalyst in the butanolysis process and implies the physical retention of sulphur-containing species during humin formation. While the presence of sulphur may be problematic for some valorisation pathways, its identification here suggests the need for post-processing strategies, such as thermal treatment, washing, or chemical modification, to remove or mitigate sulphur contamination. The environmental implications of sulphur retention are non-trivial, particularly for applications involving soil contact, catalytic reuse, or biological processing, where sulphur removal would be necessary to avoid toxicity or catalyst poisoning.

Owing to their elemental carbon richness, humins hold promise for a range of applications similar to those of biochar. Potential uses include soil amendment, carbon sequestration, and replacement of peat moss in horticultural substrates.^82,83^ These applications contribute positively to the environmental profile of biorefinery processes by valorising solid residues and reducing the overall life cycle impact. However, it is essential to note that for soil-related or environmental applications, the sulphur content must be reduced to meet agronomic and regulatory standards, as excessive sulphur can acidify soils or introduce phytotoxicity.^84^ In addition, the generation of process water during butanolysis carries implications for downstream separation and wastewater treatment, emphasising the need to integrate water management considerations into future assessments of alcoholysis sustainability.

Further research should prioritise the molecular characterisation of humins to identify structural features relevant to their reactivity, stability, and functional group availability. This will allow for the development of tailored applications for humins and ensuring they are not treated merely as waste streams, but rather as valuable co-products in lignocellulosic bioconversion pathways. Future studies should also explore purification strategies to reduce sulphur content and evaluate environmental impacts associated with humin utilisation and process water handling to support the development of fully sustainable biomass to biofuel conversion schemes.

#### 4.5.2. Carbon molar yields of humins

Table 7 presents the molar carbon yields of humins formed during the acid-catalysed butanolysis of feedstocks of varying complexity. The carbon yields are calculated assuming that all carbon in the humin fraction originates from the feedstock.

**Table 7:** Carbon yields (mol%) of humins derived from various feedstocks

Feedstock	Humins carbon content (wt%)	170 °C	190 °C	210 °C
Glucose	64.6	24.9	27.7	26.8
Cellulose	59.5	29.6	34.0	32.1
Xylan	61.9	75.1	72.0	67.7
Corn cob	48.4	30.3	33.1	35.1

Glucose and cellulose yield relatively modest average carbon contents in humins across the examined temperature range, at 26.5 and 31.9 mol%, respectively. These values are notably lower than the corresponding carbon yields to *n*-butyl levulinate, at 38.9 and 35.9 mol%, indicating that a greater fraction of feedstock carbon is converted into the desired advanced biofuel product than is lost to solid residues under the applied conditions.21
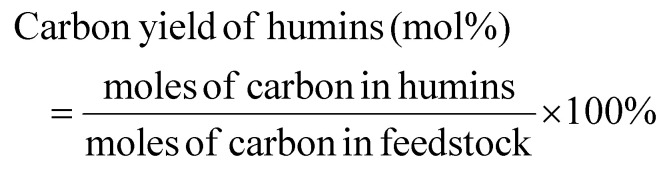


In contrast, corn cob yields humins with an average carbon yield of 32.8 mol%, while only 13.5 mol% of the carbon is recovered as *n*-butyl levulinate. This outcome is expected, as only the polysaccharide fraction (cellulose and hemicellulose), comprising approximately 56.6 wt% of the biomass, is reactive under alcoholysis conditions. The remainder, primarily lignin and extractives, contributes little to *n*-butyl levulinate formation but may promote side reactions that lead to humin formation. These findings underscore the importance of valorising humins co-produced from lignocellulosic alcoholysis, which show promise for applications as carbon-rich soil conditioners and long-term carbon sequestration materials.^73^

Xylan, a hemicellulosic pentosan polymer, yields the highest humin carbon among all feedstocks studied, averaging 71.6 mol%. As shown in Fig. 9, xylan rapidly releases furfural at early reaction stages, consistent with literature reports. Furfural is known to undergo acid-catalysed polymerisation in alcoholic solvents,^85^ contributing substantially to the mass of the resulting insoluble humin fraction. This behaviour may explain the disproportionate humin yield observed for xylan relative to other substrates.

### 4.6. Composition of product mixture

**Fig. 10:**
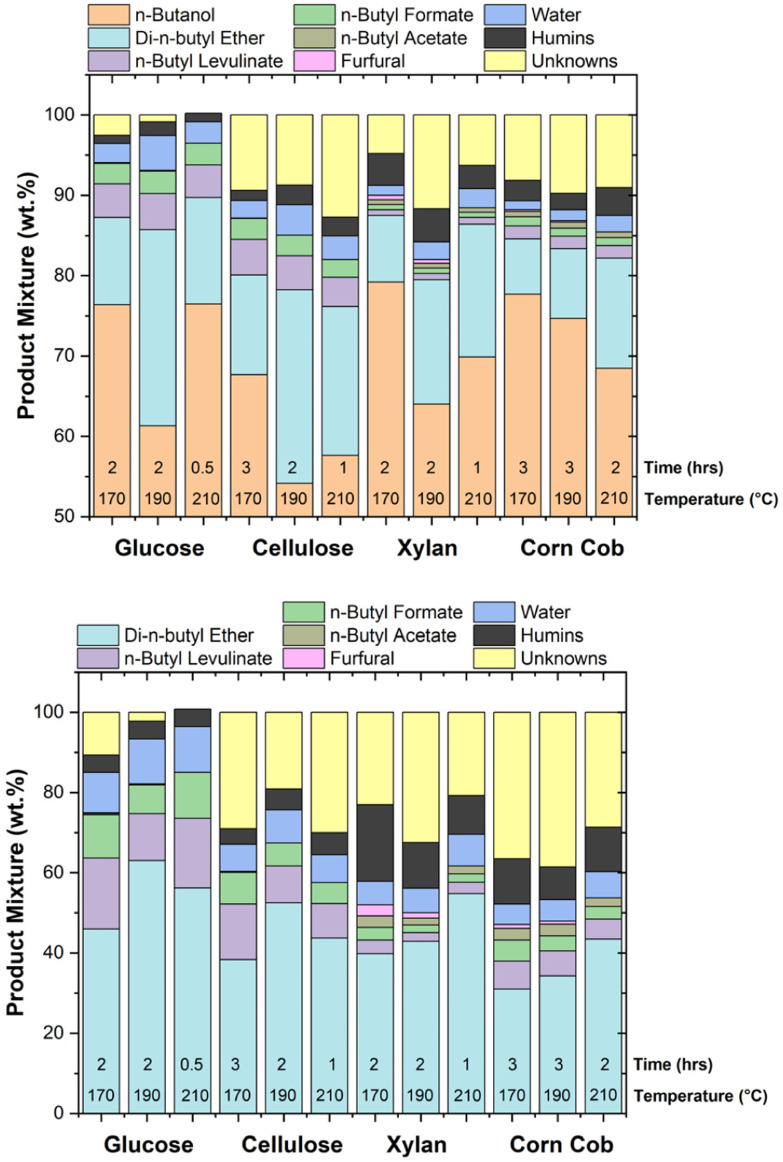
Product mixtures (wt%) for the acid-catalysed butanolysis of glucose, cellulose, xylan, and corn cob at 170, 190, and 210 °C. The time-points correspond to the shortest intervals at which steady-state was achieved for *n*-butyl levulinate formation. In panel a: the full product mixture. In panel b: the product mixture excludes *n*-butanol for better resolution of minor species.

The product mixtures from the acid-catalysed butanolysis of glucose, cellulose, xylan, and corn cob (10 wt%) at varying temperatures (170, 190, and 210 °C) and reaction times highlight the importance of optimising reaction conditions to maximise the desired *n*-butyl levulinate while minimising the formation of di-*n*-butyl ether and other by-products. Across all feedstocks, *n*-butyl levulinate mass fractions were highest for glucose and cellulose, with maximum values of 4.5 wt% of the product mixture each at lower reaction temperatures (170–190 °C). Xylan and corn cob, in contrast, showed significantly lower *n*-butyl levulinate mass fractions of 0.9 and 1.6 wt% of the product mixture, when using a feedstock loading of 10 wt%. This is attributed to their lower glucan content and more complex structures. Fig. 10 shows the full product mixtures (wt%) as well as a focused view excluding *n*-butanol for improved resolution of minor species. Although higher masses of *n*-butyl levulinate in the product mixture are desirable, achieving them can be challenging. Preliminary experiments using feedstock loadings of 20 wt% resulted in highly viscous product mixtures, with higher humin concentrations and insufficient supernatant to allow for accurate and reliable determination of product concentrations. Therefore, moderate feedstock loadings are recommended for butanolysis reactions involving lignocellulosic biomass.

#### 4.6.1. Identification of minor species

In addition to the primary products, *n*-butyl levulinate, *n*-butanol, and di-*n*-butyl ether, *n*-butyl formate was identified in the product mixtures from all four feedstocks examined in this study. *n*-Butyl formate, which forms *via* the esterification of formic acid and butanol, was detected in notable concentrations, reaching up to 74 wt% relative to *n*-butyl levulinate. Formic acid is a well-established by-product of ethyl levulinate formation, as reported in previous studies,^22,23,41^ and is likewise expected to form under analogous butanolysis conditions. Its presence in the system therefore provides a clear mechanistic route for the formation of *n*-butyl formate, as illustrated in reaction 6 in Table 2.

*n*-Butyl acetate and furfural were also identified in the product mixtures from xylan and corn cob. Their formation is attributed to the acetyl groups present in these feedstocks, as discussed in Section 4.1.3, and to the depolymerisation and subsequent dehydration of xylan monomers, which generate furfural at short reaction times. All species identified were confirmed through mass spectrometry. Their formation pathways are incorporated into the surrogate kinetic model through a simplified reaction scheme designed to account for minor side reactions.

#### 4.6.2. Reaction mixture composition

A critical observation is the trade-off in formation between *n*-butyl levulinate and di-*n*-butyl ether. Higher temperatures and longer reaction times favour di-*n*-butyl ether production, particularly for glucose and cellulose, with mass fractions reaching 24.4 and 24.1 wt% at 190 °C, respectively, even at the earliest steady-state conditions of levulinate production. It is important to note that the reported mass fractions reflect the composition of these products in the reaction mixture rather than their actual yields. This underscores the need to optimise reaction conditions to maximise *n*-butyl levulinate formation while limiting di-*n*-butyl ether production, which not only consumes valuable alcohol but also reduces atom economy and process sustainability. Notably, di-*n*-butyl ether generation contributes to the water content of the reaction mixture, with mass fractions reaching up to 4.3 wt% (glucose at 190 °C). The presence of water complicates downstream separation and can shift equilibria unfavourably, promoting reverse hydrolysis of esters and lowering overall levulinate yields. Therefore, minimising water formation, through careful control of reaction pathways or integrating water removal strategies, represents a critical target for improving the efficiency and green metrics of the butanolysis process.

#### 4.6.3. Value of by-products

While di-*n*-butyl ether is generally considered an undesired by-product in this context, it holds potential as an additive for diesel fuel. When used at controlled concentrations, it improves cold flow properties and enables the formulation of blends that meet fuel property standards such as flash point, density, and viscosity.^20^ Therefore, at moderate concentrations, it can enhance the overall atom efficiency of the butanolysis process, positioning it as a valuable co-product.

Conversely, *n*-butyl acetate is utilised across multiple sectors. It serves as a solvent in the production of adhesives, lacquers, coatings, and paints, contributing to the manufacture of consumer products such as automobiles and wood furniture. In the pharmaceutical and cosmetic industries, it functions as an extraction agent or solvent, while in the food industry, it is employed to synthesize fruit flavours. Additionally, *n*-butyl acetate has been explored as a biofuel additive, with studies indicating that its inclusion in biodiesel blends can reduce soot and greenhouse gas emissions without adversely affecting combustion heat and cetane number.^86,87^

Moreover, *n*-butyl formate has been investigated for its potential as a biofuel, with studies examining its combustion behaviour, such as ignition delay and laminar flame speed, highlighting its suitability as an alternative fuel component.^88^ Additionally, its occurrence in fruits like apples and strawberries highlights its potential application in the flavour and fragrance industries.^89^ These diverse uses underscore the economic value of *n*-butyl formate as a by-product of the process.

Furfural, derived from lignocellulosic biomass, serves as a versatile platform chemical with numerous industrial applications. It is widely used to produce resins and adhesives, particularly within the plastics and coatings industries.^90^ In the pharmaceutical sector, furfural acts as a precursor for various medicinal compounds, while in agriculture, it contributes to the synthesis of herbicides and nematicides.^91^ Additionally, furfural functions as a selective solvent in refining processes, particularly for lubricating oils and the extraction of dienes in synthetic rubber manufacture. However, under the acidic, moderate-temperature conditions typical of butanolysis, furfural may also undergo polymerisation, contributing to the formation of humins. These insoluble carbonaceous by-products may contain these furanic structures. Thus, while furfural valorisation presents a promising route to enhance process economics, its role in humin formation must also be considered in the context of product yield and carbon efficiency, but is beyond the scope of this paper.

This analysis highlights the potential for refining the reaction system to selectively enhance the mass fraction of *n*-butyl levulinate while simultaneously leveraging by-product valorisation strategies. These efforts, combined with the predictive power of the surrogate kinetic model, can improve the sustainability and profitability of advanced biofuel production. By designing reaction systems that maximise desired products while valorising side products, the prevention of waste is targeted in alignment with Green Chemistry principles. Given its impact on both thermodynamic equilibrium and process economics, water generation should also be considered a key optimisation target, alongside humin suppression and alcohol preservation, in future butanolysis system design. Identifying valuable co-products such as *n*-butyl acetate, di-*n*-butyl ether, and furfural exemplifies how comprehensive systems analysis can reveal opportunities for integrated biorefinery designs, further enhancing environmental and economic sustainability.

### 4.7. Hierarchical biochemical surrogate kinetic model

#### 4.7.1. Developments from previous models

The fourth-generation surrogate kinetic model presented here builds upon earlier versions developed by McNamara^22^ and O'Shea *et al.*,^23^ incorporating several key advancements to improve predictive capability and mechanistic accuracy.

First, the model has been newly trained using acid solvolysis data obtained in *n*-butanol solvent systems, enabling its application to both ethanolysis and butanolysis reactions.

Second, the model has been extended to predict the yields of all non-trace products in the reaction mixture, including *n*-butyl levulinate, di-*n*-butyl ether, butanol, furfural, butyl acetate, formic acid, butyl formate, humins, and water.

Third, the model ensures strict mass conservation with the unaccounted mass categorised as “soluble unknowns”, representing a diverse array of trace species solubilised in the reaction medium. Distinct compositions of soluble unknowns are associated with each feedstock: glucose, cellulose, xylan monomer, acetylated xylan monomer, furfural, and residual corn cob biomass (comprising lignin, ash, extractives, and other unidentified constituents). While the precise molecular identities of these soluble unknowns are unresolved, their grouped elemental composition provides insight into the likely classes of trace species present. For every humin-forming pathway, a corresponding route to a unique soluble unknown composition is included, thereby ensuring overall mass conservation.

Fourth, each pathway leading to the formation of humins, or soluble unknowns, is represented by two parallel reactions: one catalytic (regenerating hydrogen ions) and one non-catalytic (consuming hydrogen ions). This structure captures the acid consumption phenomenon observed in the acid-catalysed alcoholysis of lignocellulosic biomass, as reported by McNamara *et al.*^22^

Finally, the kinetic parameters, including pre-exponential factors, activation energies, and rate constants, are summarised in Table 2. These were optimised using the algorithm described in Section 3.3.4.

#### 4.7.2. Novel product predictions: furfural, *n*-butyl acetate, and *n*-butyl formate

The surrogate kinetic model successfully predicts the concentrations of all six major value-added products formed in the butanolysis reaction mixture. In particular, the early formation of furfural and its subsequent decomposition to insoluble solid residues (humins) is well replicated. The formation profile of *n*-butyl formate is also accurately captured by the model.

Among the three novel products, *n*-butyl acetate presents the greatest modelling challenge, particularly when applying the surrogate model to the complex corn cob feedstock. In the model, *n*-butyl acetate formation is described by an esterification reaction between an acetylated xylan monomer and a molecule of *n*-butanol, yielding one molecule of *n*-butyl acetate and a deacetylated xylan monomer. The degree of xylan acetylation, 33%, was quantified by NMR spectroscopy and is reported in Section 4.1.3. Accordingly, xylan is represented with one acetyl group per trimer.

When isolated xylan was used as the feedstock, the predicted *n*-butyl acetate concentrations closely matched the experimental data. However, corn cob biomass produced comparable concentrations of *n*-butyl acetate despite xylan comprising only approximately 24.2 wt% of the material. This behaviour was unexpected, as the *n*-butyl acetate yield would be anticipated to scale with the xylan content alone. These results suggest that either the xylan present in the untreated corn cob possesses a higher degree of acetylation than measured for the isolated xylan standard or, more plausibly, that other acetyl-containing constituents within the corn cob, particularly lignin-derived moieties, contribute to ester formation under the reaction conditions.

Consequently, the model underpredicts *n*-butyl acetate yields for the corn cob system, as esterification is currently attributed exclusively to xylan-derived acetyl groups. This limitation indicates that associated lignin-acetylated structures and other non-carbohydrate contributions to ester formation are not yet incorporated within the kinetic framework. Future refinement of the surrogate model will involve extending the esterification reaction network to capture these additional pathways and improve prediction accuracy for chemically complex lignocellulosic substrates.

#### 4.7.3. Hierarchical biochemical surrogate kinetic model

Fig. 9 presents the concentration–time profiles of the principal liquid products obtained from butanolysis, alongside predictions from the surrogate kinetic model. The model demonstrates strong agreement with the experimental data across most species, accurately replicating the behaviour of *n*-butanol (*R*^2^ = 0.99), di-*n*-butyl ether (*R*^2^ = 0.91), *n*-butyl levulinate (*R*^2^ = 0.92), and *n*-butyl formate (*R*^2^ = 0.94). Similarly, the concentration of *n*-butyl acetate is predicted with high fidelity for model substrates (*R*^2^ = 0.95), although notable deviations arise in the case of corn cob. As previously discussed, this discrepancy reflects the release of acetyl groups from both hemicellulose and lignin, which are not fully incorporated into the current model configuration (Table 8).

**Table 8:** Coefficients of determination (*R*^2^) for the major butanolysis products

	A*n*-Butanol	Di-*n*-butyl ether	*n*-Butyl levulinate	*n*-Butyl formate	*n*-Butyl acetate	Furfural
*R* ^2^	0.99	0.91	0.92	0.94	0.95	0.62

The weakest correlation was observed for furfural (*R*^2^ = 0.62), highlighting the challenge of fully capturing hemicellulose degradation and the subsequent side reactions responsible for early-stage humin formation. This reduced accuracy indicates that key mechanistic pathways associated with xylose dehydration, furfural reactivity, and condensation to humins are not yet comprehensively represented in the current model. Ongoing experimental studies using isolated xylose and furfural feedstocks are being undertaken to elucidate these reactions, and the resulting data will support the expansion of the kinetic network to strengthen predictive capability.

These outcomes are further illustrated in Fig. 10, which depicts the complete composition of the product mixtures under steady-state conditions. Taken together, these results demonstrate that the butanolysis hierarchical surrogate kinetic model provides a reliable and practical framework for evaluating biomass butanolysis systems. By enabling quantitative prediction of value-added liquid products based solely on the biochemical composition of the feedstock, the model offers significant utility for both life cycle and techno-economic assessments. Such predictive capability facilitates rational process optimisation and feedstock prioritisation, thereby accelerating the commercial readiness of alcoholysis technologies.

Crucially, the model supports the strategic assessment of diverse lignocellulosic resources, enabling efficient allocation of biomass streams to maximise product yields and carbon efficiencies. The resulting three-component fuel mixtures have demonstrated compatibility with both diesel and gasoline engines, with stable blending at concentrations up to 25%.^20^ These findings underline the potential of alcoholysis-derived fuel blends to reduce greenhouse gas emissions across passenger and heavy-duty transport sectors. Championing scale-up and deployment of this pathway, underpinned by robust mechanistic modelling, is therefore vital to advancing transport decarbonisation objectives.

#### 4.7.4. Predicted space-time yields at *T*_90_

The hierarchical biochemical surrogate kinetic model was used to estimate yields of *n*-butyl levulinate and di-*n*-butyl ether across various feedstocks and temperatures. As a performance metric, space-time yield quantifies the mass of product formed per unit reactor volume per unit time. It is commonly used to assess process productivity and scalability in chemical engineering.^48,92,93^ In this study, space-time yield was calculated at the time required to reach 90% of the maximum predicted *n*-butyl levulinate yield (*T*_90_), which was defined as a practical steady-state condition. Space time yields and associated product distributions at *T*_90_ for all feedstocks and reaction temperatures are summarised in Table 9.

**Table 9:** Hierarchical biochemical surrogate kinetic model predictions

Feedstock	Reactor size (mL)	Temp. (°C)	Max. BL yield (g)	0.9 × BL (g)	*T*_90_ (h)	*n*-DBE yield at *T*_90_ (g)	Humin yield at *T*_90_ (g)	Space-time yield (g L^−1^ h^−1^)
Glucose	17.5	170	0.221	0.199	1.4	0.45	0.08	8.4
		190	0.220	0.198	0.9	0.70	0.08	13.3
		210	0.218	0.196	0.6	0.98	0.08	19.2
Cellulose	17.5	170	0.199	0.179	2.1	0.71	0.10	4.9
		190	0.193	0.174	1.5	1.06	0.10	6.9
		210	0.186	0.168	1.0	1.52	0.11	9.4
Xylan	17.5	170	0.042	0.038	1.1	0.32	0.17	2.0
		190	0.042	0.038	0.6	0.39	0.17	3.7
		210	0.042	0.038	0.4	0.44	0.17	5.6
Corn cob	17.5	170	0.063	0.057	3.1	0.54	0.09	1.1
		190	0.064	0.058	2.2	0.87	0.10	1.5
		210	0.063	0.056	1.7	1.31	0.10	2.0

Higher temperatures consistently reduced *T*_90_, due to faster reaction kinetics. However, this acceleration came with a trade-off: increased di-*n*-butyl ether formation at higher temperatures, even at the reduced *T*_90_ reaction times. For example, in the glucose system, raising the temperature from 170 °C to 210 °C decreased *T*_90_ from 1.4 h to 0.6 h and increased space-time yield from 8.4 to 19.2 g L^−1^ h^−1^, while the di-*n*-butyl ether yield more than doubled (from 0.45 to 0.98 g), a side reaction that not only consumes the costly alcohol but also generates water, which must be removed. This selectivity–intensification trade-off was observed across all feedstocks studied, with varying magnitudes. Notably, the quantity of humins isolated at *T*_90_ remained consistent across temperatures, indicating that shorter reaction durations at higher temperatures do not significantly affect the extent of humin formation.

A note on reactor scale and configuration is warranted. The present study employs a small-scale batch reactor to allow precise control over reaction conditions and to support mechanistic interpretation. As such, the reactor system was not designed to explore engineering parameters such as heat and mass transfer limitations, residence time effects, catalyst contacting efficiency, or continuous flow operation. A full optimisation of reactor design and scale-up behaviour falls outside the scope of this work but is the focus of ongoing collaborative studies. These future investigations will evaluate how reactor architecture influences space-time yield, selectivity, and overall process efficiency, thereby supporting translation of the butanolysis system toward industrial deployment.

These findings highlight the need to carefully balance conversion rate and selectivity when optimising reaction conditions. A detailed techno-economic analysis will be required to evaluate whether the gains in space-time yield at higher temperatures are sufficient to offset the increased formation of side products and their associated separation and environmental costs.

## 5. Conclusions

We have systematically evaluated the acid-catalysed butanolysis as a viable thermochemical pathway to produce *n*-butyl levulinate, a promising advanced biofuel and platform chemical, utilising diverse biomass-derived feedstocks. By investigating glucose (a monosaccharide), cellulose and xylan (key polysaccharide components), and corn cob (real-world lignocellulosic biomass), the influence of feedstock composition on reaction efficiency, conditions, and product distribution was determined.

*n*-Butyl levulinate yields were found to vary depending on the feedstock type, with maximum yields of (49.6 ± 2.7) mol% for glucose at 170 °C, (43.4 ± 0.7) mol% for cellulose at 190 °C, (28.8 ± 3.1) mol% for corn cob at 210 °C, and (8.9 ± 3.7) mol% for xylan at 210 °C. These differences highlight the variability in conversion efficiency, underscoring the need to tailor reaction conditions to the structural and compositional characteristics of each substrate. While elevated temperatures facilitated depolymerisation and sugar release, they also fostered side reactions, underscoring the trade-off between maximising *n*-butyl levulinate yields and minimising by-products. Steady-state times tended to increase with both feedstock complexity and decreasing reaction temperatures, reflecting the kinetic barriers posed by the lignocellulose matrix. Shorter reaction times minimised di-*n*-butyl ether formation, illustrating the importance of identifying the earliest steady-state point for optimal techno-economic outcomes.

Because *n*-butyl levulinate originates from both cellulose and hemicellulose fractions, reporting yields based solely on cellulose content can significantly overestimate process efficiency. This study, therefore, adopts a more representative method: molar yields are calculated relative to the theoretical maximum based on the total cellulose and hemicellulose content. This approach enables more accurate benchmarking of lignocellulosic feedstock performance and is proposed as a standard for future reporting of molecular product yields from complex biomass sources.

Minor by-products, including *n*-butyl formate, *n*-butyl acetate, and furfural, were consistently detected in the butanolysis of xylan and corn cob. While these compounds typically appear in lower quantities, their concentrations can become significant under optimal conditions for the production of *n*-butyl levulinate. For instance, *n*-butyl formate reached 74 wt% and *n*-butyl acetate 41 wt% relative to levulinate formation in the case of corn cob at the earliest steady-state conditions. These by-products, often overlooked in the literature, may have substantial implications for the economic viability of the process, highlighting the need for careful quantification and potential valorisation as solvents, chemical intermediates, or fuel additives. The detection of *n*-butyl acetate in product mixtures derived from both xylan and corn cob indicated the presence of acetyl groups within the hemicellulose fraction of the biomass. This observation was corroborated by nuclear magnetic resonance analysis, which quantified a mean monomer-based degree of acetylation of 0.34.

The hierarchical biochemical surrogate kinetic model developed herein builds upon prior iterations by incorporating novel features such as mass-conserving product pathways, catalytic and non-catalytic humin formation mechanisms, and grouped soluble unknown species to account for trace mass. The model successfully predicted the yields of all major and minor products, including furfural, *n*-butyl formate, and *n*-butyl acetate. Notably, the model highlighted a key discrepancy in *n*-butyl acetate prediction from corn cob feedstock, suggesting that acetylated moieties within the non-carbohydrate constituents of the biomass, such as lignin, may also contribute to ester formation, an area warranting further investigation. By enabling accurate product yield prediction from biomass composition alone, the model provided a valuable tool for life cycle assessment, techno-economic analysis, and process optimisation. It also facilitated strategic feedstock screening to maximise carbon efficiency and supported the design of scalable systems capable of producing three-component biofuel mixtures (alkyl levulinate, dialkyl ether, and alcohol), which are compatible with diesel and gasoline engines at up to 25 vol% blends.^20^ These findings have direct relevance to the decarbonisation of both light-duty and heavy-duty transport sectors.

This study demonstrated that one-pot butanolysis of lignocellulosic biomass can be tuned to selectively produce *n*-butyl levulinate and other high-value co-products, with viable yields and robust kinetic modelling support. Industrial scalability will depend on continued efforts in catalyst recovery, continuous-flow processing, and valorisation of all carbon-containing fractions, including humins. Our fourth-generation surrogate kinetic model can provide a strong foundation for future simulation-driven optimisation, ultimately enabling more efficient and economically attractive routes to renewable fuel production in an integrated biorefinery context.

## Author contributions

Conall McNamara, Ailís O'Shea, and Stephen Dooley conceived and designed the study. Conall McNamara carried out the investigation, with data curation performed by Conall McNamara and Tiarnán Watson-Murphy. Formal analysis was undertaken by Conall McNamara, Ailís O'Shea, Thiago De Melo Lima, and Leandro Ayarde-Henríquez. The work was supervised by Stephen Dooley. The original draft of the manuscript was prepared by Conall McNamara. All authors contributed to reviewing and editing the final version.

## Conflicts of interest

There are no conflicts to declare.

## Data Availability

All data supporting the findings of this study are contained within the article. Additional datasets, including unprocessed GC-MS outputs, kinetic model input files, raw NMR spectra, and supporting calculations, contain proprietary information associated with ongoing industrial collaborations and therefore cannot be made publicly available. These materials can be shared with qualified researchers upon reasonable request and subject to appropriate confidentiality agreements.
